# Chiral molecular 4f qubits by post functionalization

**DOI:** 10.1039/d5qi00977d

**Published:** 2025-09-01

**Authors:** Steen H. Hansen, Christian D. Buch, Bela E. Bode, Stergios Piligkos

**Affiliations:** a Department of Chemistry, University of Copenhagen Universitetsparken 5 2100 Denmark piligkos@chem.ku.dk; b EaStCHEM School of Chemistry, Biomedical Sciences Research Complex, and Centre of Magnetic Resonance, University of St Andrews North Haugh St Andrews KY16 9ST UK beb2@st-andrews.ac.uk

## Abstract

We herein demonstrate the synthesis of a pair of enantiomerically pure Yb^III^ complexes by post-functionalisation of the parent Yb^III^ complex *via* condensation with an enantiomerically pure chiral amine. The enantiomeric pair is structurally characterised by single crystal and powder X-ray diffraction, showing that it crystalises in the *P*2_1_2_1_2_1_ Sohncke space group with Flack parameters close to zero, which confirms their enantiopurity. Circular Dichroism (CD) and absorption spectroscopies in the NIR reveal sharp ^2^F_7/2_ → ^2^F_5/2_ f–f transitions, with *g*_abs_ values up to 0.07, indicating a chiral environment for the ytterbium centre. Furthermore, a dynamic mechanism with mixing of ligand states is shown to contribute to the CD intensity. X-band pulse Electron Paramagnetic Resonance spectroscopy, on a magnetically dilute single crystal containing 1% of Yb^III^ complexes within the isostructural Y^III^ diamagnetic host, reveals a phase memory time, *T*_m_, of the electronic spin of 600 ns and that it can be coherently manipulated by microwave pulses, as evidenced by Rabi nutations.

## Abstract 2

A pair of enantiomerically pure Yb^III^ complexes are synthesised and pulse EPR Rabi nutations and phase memory times show the complexes are suitable for coherent spin manipulation.

## Graphical abstract

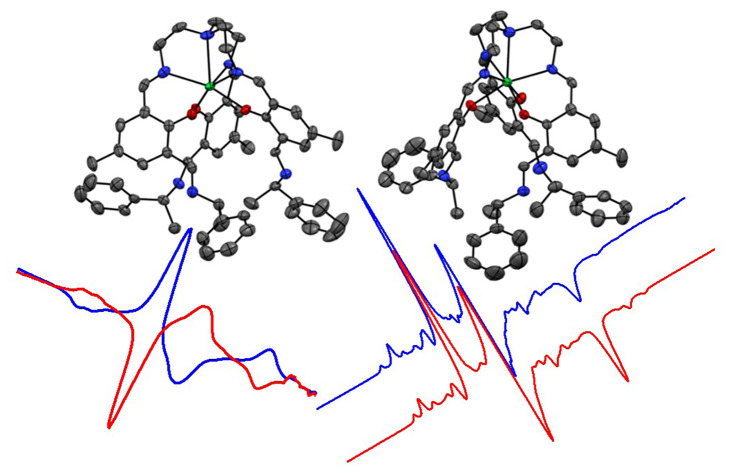

## Introduction

Coherent manipulation of electron spins has been proposed as the foundation of various technological applications within the broad context of Quantum Technologies (QTs).^1,2^ QTs encompass technologies that exploit fundamental quantum mechanical properties of matter such as superposition and entanglement to obtain superior performance, previously unattainable by classical counterparts.^3^ These new technologies are of interest for a broad range of applications, ranging for example from quantum computers^4^ to highly sensitive sensors.^5^ Molecules have been proposed as quantum hardware due to several attributes which make them promising qubits or qudits, such as their long coherence times at high temperatures,^6^ their tunability and diversity, allowing for a near-infinite number of possibilities for these materials.^7–11^ Yb(trensal), is a trigonal lanthanide coordination complex,^12^ which was recently shown to possess a suitable electron spin phase memory time, *T*_m_, and the ability to be coherently manipulated, indicating its potential as an electron qubit.^13^ Implementation of quantum error correction protocols was demonstrated using the hyperfine coupling of the electronic spin to the ^173^Yb nucleus^14^ and this coupling was later used for the implementation of a quantum simulator on the electronuclear qudit.^15^ Furthermore, dipolar coupled Yb^III^ sites could be coherently manipulated, demonstrating quantum gates between two dipolarly coupled entangled qubits.^16^

**Fig. 1:**
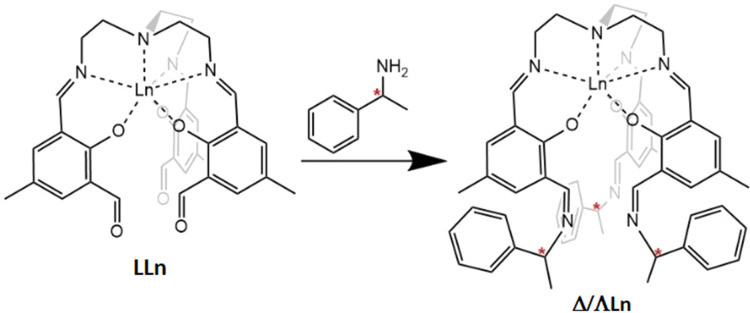
Reaction scheme for post-functionalisation of LLn with methyl-benzylamine. Red asterisks indicate the stereogenic centre.

Coherently addressing and manipulating the state of a spin qubit, such as a molecular spin, is typically achieved by use of magnetic dipole transitions induced by the oscillating magnetic field component of microwave pulses in an externally applied magnetic field.^17^ However, a very interesting alternative avenue is the potential substitution of microwave pulses with the application of electric fields coupled to the spin qubit,^18^ the magnetic dipole transition in this case being mediated by the magneto-electric coupling.^19^ Furthermore, electric fields can be used to tune the resonance frequency of molecular qubits and could be used to selectively bring specific qubits on and off resonance within multiqubit processor setups.^20^ The main advantages of using electric rather than magnetic fields include the ability to control electronics on a nanosecond timescale, the highly precise electronics instrumentation already developed and the very power-efficient operation of electronic circuits.^21–23^ Efficient coupling of molecular spins to electric fields requires absence of an inversion centre at the molecular level.^20,24^ Hence, designing new chiral molecules with relevant properties for quantum information processing or other QTs is of great interest.^25^ Since Yb(trensal) has been shown to display numerous properties of interest for QTs, an enantiomeric pure analogue of Yb(trensal) would provide an interesting avenue for studying chiral effects related to quantum information processing. From a preparative perspective, there is only a very limited number of options available for the synthesis of enantiomerically pure lanthanide complexes where chirality is due to the helicity of the coordination sphere environment. This is due to the very small stabilisation effect of the ligand field, resulting in near instantaneous racemization in these complexes in solution.^26^ Therefore, the synthesis of chiral lanthanide complexes often involves reacting a simple lanthanide salt or nonchiral coordination complex with a previously synthesized enantiomerically pure ligand,^27–35^ or by precipitation of an ionic complex with a chiral counterion.^27^ We have previously shown that post-functionalization of the complex LLn (Fig. 1), with Ln = Gd, Tb, Dy, Ho, Er, Tm, Yb or Lu, with primary amines provides a direct route towards adding desired properties to Ln-based coordination complexes.^36^ In these previous studies we have only used non-chiral amines.^37,38^ Herein, we demonstrate that condensation of LYb with an enantiomerically pure primary amine (*S*- or *R*-methyl-benzylamine) provides the means to form chiral ytterbium complexes exhibiting coordination sphere helicity (ΔYb or ΛYb), an avenue that has not been previously explored for trensal-like ligands. These studies affirm the versatility of our post-functionalization approach to synthesize functional coordination compounds. The chiroptical properties of the synthesized ytterbium complexes are examined, and their origin investigated. Furthermore, these chiral materials are examined by a combination of continuous wave (c.w.) and pulse Electron Paramagnetic Resonance (EPR) spectroscopy demonstrating that they retain a suitable phase memory time and the ability of their electronic spin to be coherently manipulated by microwave pulses.

## Experimental section

The parent complex, LYb, was synthesised according to literature procedures.^36^ All reagents were purchased from commercial sources and used as received.

### Synthesis of ΔYb/ΛYb

LYb (200 mg, 0.265 mmol) was suspended in a 1 : 1 chloroform/methanol solution (20 ml) and *S*- or *R*-methyl-benzylamine (0.4 ml, 3 mmol) was added resulting in ΔYb or ΛYb, respectively (*vide infra*). The reaction was stirred for 1 hour after which a clear solution was obtained. 200 ml diethylether were added to the solution which was covered with a glass cover and left to crystalize for 2–4 days.

Yield: around 160 mg, 58%. Compositional and phase purity was confirmed by elemental analysis (Table S1), IR (Fig. S11 and S12), and X-ray powder diffraction (Fig. S15).

^1^H-NMR (Fig. S16–S18) was obtained using a Bruker 500 MHz instrument equipped with a cryoprobe. For ^1^H-NMR calibration was done against solvent signals from the deuterated solvent. Positive-ion mode MALDI mass spectrometry (Fig. S19–S22) was performed on a Bruker Solarix XR 7T ESI/MALDI FT-ICR MS instrument at the Department of Chemistry, University of Copenhagen. Elemental (C, H, and N) analyses were performed on a FlashEA 1112 instrument at The Microanalytical Laboratory at the Department of Chemistry, University of Copenhagen.

Single crystals were measured on a BRUKER D8 VENTURE diffractometer equipped with a Mo Kα High-brilliance IμS 53 radiation source (*λ* = 0.71073 Å). A PHOTON 100 CMOS detector was employed. The instrumentation was controlled using APEX2. The sample was cooled to 120 K using an Oxford cryosystem. The resulting data were modelled using SHELXT with intrinsic phasing and refined using SHELXL (Least squares).^39^ Visualisation of the refinement process was done using OLEX2.^40^ Hydrogens were added using the “Add H” function in Olex2 and refined isotopically, while all non-hydrogen atoms were refined anisotropically.^40^

Absorption spectroscopy was performed on a PerkinElmer Lambda 2 UV/Vis spectrometer with a 2 nm slit width. Circular dichroism (CD) was measured on a Jasco J1700 equipped with an InGaAs detector for NIR with a 5 nm slit. Both Absorption and CD (Fig. 3, S13 and S14) were measured in approximately 15 mg ml^−1^ solutions in chloroform in 10.0 mm path quartz cuvettes. Absorption and CD were measured back to back on the same cuvette. Infrared spectra were measured on polycrystalline samples on an Agilent Technologies Cary 630 FTIR.

EPR spectra were measured on a magnetically dilute sample of ΛYb in the isostructural diamagnetic ΛY host lattice at a concentration of 1% (**ΛY**_**0.99**_**Yb**_**0.01**_), as determined by Inductively Coupled Plasma Mass Spectrometry (ICP-MS). X-band c.w.-EPR data were measured on a polycrystalline powder sample using a BRUKER E500 EPR spectrometer fitted with a Bruker X-band ER4122 SHQE cavity resonator. The measurements were performed at 20 K using an Oxford Instruments helium flow cooling system.

Single crystal pulse EPR was measured on a BRUKER X-band E580 EPR spectrometer fitted with an MS3 resonator appropriate for smaller samples and offering higher *B*_1_ excitation fields, on single crystals of approximate size 0.2 × 0.2 × 3 mm.^3^ A cryogen free variable temperature cryostat from Cryogenics Ltd was used. Echo-Detected Field-Swept (EDFS) EPR spectra were measured using a standard Hahn echo sequence (π/2–*τ*–π–*τ*-echo) with π/2 = 16 ns. *T*_m_ was measured by recording the time evolution of the Hahn echo, with π/2 = 128 ns to decrease ESEEM (electron spin echo envelope modulation). Spin lattice relaxation, *T*_1_, was measured using a standard inversion recovery sequence (π–*T*–π/2–*τ*–π–*τ*-echo) with π/2 = 16 ns. Both *T*_1_ and *T*_m_ were modelled as mono-exponential decays. Transient nutation was measured using a *θ*–*T*–π/2–*τ*–π–*τ*-echo sequence with *θ* the nutation pulse angle and *T* = 5000 ns, at 6 different power levels between 6–18 dB.

The c.w.- and EDFS EPR spectra were fitted using Pepper in Easyspin (version 6).^41^ EDFS spectra can be assimilated to zero-th derivative c.w. spectra in cases where *T*_m_ is the same for all observed transitions and ESEEM or other dynamic modulation effects affecting individual echo intensities are negligible. The single crystal orientation was determined by fitting the sample orientation and molecular frame using the spin Hamiltonian parameters obtained for the polycrystalline powder.

## Results and discussion

**Fig. 2:**
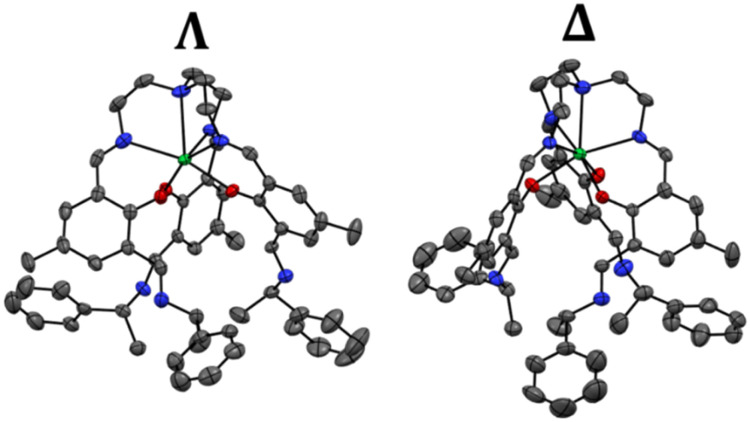
Crystal structure of ΔYb and ΛYb seen from the side showing the different handedness of the structures. Colour code: Yb (green), N (Blue), O (red). Hydrogen atoms were omitted for clarity.

Reaction of the previously studied complex, LLn with enantiomerically pure *S*- or *R*-methyl-benzylamine *via* a Schiff-base reaction in a mixture of chloroform and methanol results in the formation of ΔYb or ΛYb, respectively, depending on the chirality of the amine (Fig. 1). The resulting complexes crystalize as planks upon addition of diethyl ether, resulting to single crystals suitable for single crystal X-ray diffraction. Single crystal X-ray diffraction revealed that both enantiomers crystallise in the triclinic Sohncke space group *P*2_1_2_1_2_1_ (Table S2). The crystal structure (Fig. 2) reveals that the ytterbium ion is heptacoordinated by three phenoxides, three imines and a tertiary amine. A pseudo (non-crystallographic) threefold axis passes through the tertiary amine and the Ln-centre. This pseudo-*C*_3_ axis is very close to being ideal with bond lengths and angles only diverging slightly from trigonal symmetry. The imine (N_imi_–Ln) and phenoxide (Ln–O) bond lengths vary in the range 2.395(4)–2.429(4) Å and 2.154(4)–2.159(4) Å, respectively. ΔYb or ΛYb show very comparable bond lengths and angles to LLn (Table 1 and Tables S3–S6) indicating only very minor perturbation of the Ln environment, as was also seen in previous post-functionalisation experiments employing LYb.^36–38^ The three chiral methyl-benzylamine arms have their methyl groups pointing towards the central pseudo-*C*_3_ axis of the molecule while their phenyl groups point outward. The asymmetric unit contains the whole molecule, resulting in four identical molecules in the unit cell (Fig. S1–S8). These four units all share the same helicity around the Ln centre, which is dictated by the chiral amine with the *S*-methyl-benzylamine leading exclusively to a Δ helicity around the lanthanide centre while *R*-methyl-benzylamine leads to a Λ one (Fig. S9 and S10). Hence the solid-state structure exclusively contains one helicity, which has not been observed for any previous Ln(trensal)-like complex where both enantiomers co-crystalize resulting to crystals containing both enantiomers.

**Table 1:** Comparison of selected average bond lengths and bond angles of LYb^36^ and the chiral post functionalized complexes ΔYb/ΛYb

	N_api_–Ln/Å	N_imi_–Ln/Å	O–Ln/Å	(N_api_–Ln–O)/°
LYb	2.609(3)	2.420(2)	2.160(2)	127
ΔYb/ΛYb	2.597(3)	2.412(2)	2.156(2)	125

**Fig. 3:**
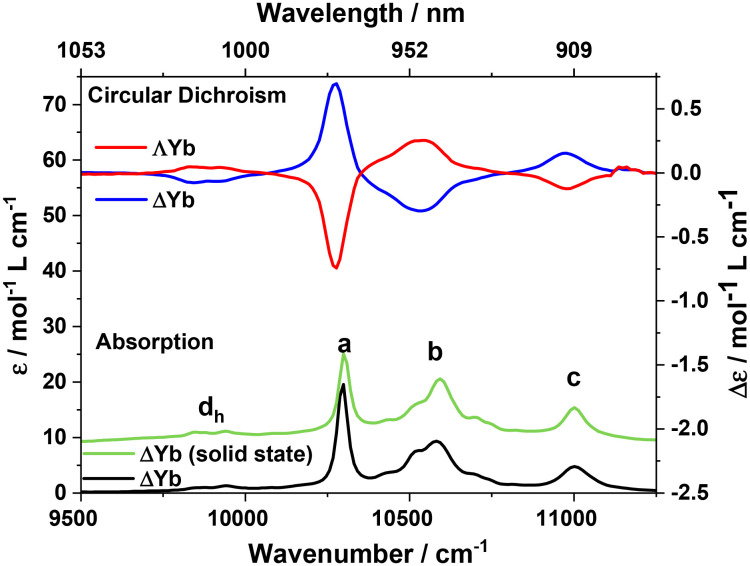
Absorption and CD spectra of ΔYb and ΛYb in chloroform. A solid-state absorption spectrum of ΔYb is also shown. Labels are defined in the main text.

Chiral molecules interact differently with left and right circularly polarised light because they break space parity symmetry. ΔYb or ΛYb display several absorption bands between 900–1050 nm belonging to the ^2^F_7/2_ → ^2^F_5/2_ transition, with the three major bands (marked **a**–**c** in Fig. 3) displaying fine structure which is assigned as vibrational fine structure (Fig. 3). The intensity of these transitions is very weak (*ε* < 20 L mol^−1^ cm^−1^) which is characteristic for lanthanides.^42^ Two minor absorption bands (marked d_h_) are assigned as hot-bands due to their emergence at lower energy and being of very weak intensity, in agreement with previous observations for Yb(tensal).^12^ The nearly identical absorption spectra in the solid state and in solution indicate dissolution of complexes without major structural changes or decomposition. This is supported by ^1^H-NMR (Fig. S16–S18) and MALDI-MS (Fig. S19–S22). The CD spectra of the ^2^F_7/2_ → ^2^F_5/2_ transitions are mirror images of each other with altering phase for various transients. The largest CD signal is observed at transition **a** which shows a Δ*ε* = 0.7 L mol^−1^ cm^−1^ and the largest dissymmetry factor, *g*_abs_ = 0.07. Although the theoretical maximum absolute value of *g*_abs_ is 2, typical values for chiral organic molecules or transition metal complexes lie in the range 10^−4^ to 10^−2^, with lanthanide complexes displaying in general values above this range, sometimes even close to the theoretical maximum.^43–45^ Thus, the dissymmetry displayed by ΔYb or ΛYb is sizeable but not exceptional for f–f transitions. This sizeable dissymmetry indicates that the relevant Yb centre feels a chiral electric potential. Thus, a strong magneto-electric response to electric fields is possible. The origin of the CD signal can either be *via* a static (mixing with ytterbium-based 5d orbitals) or dynamic (mixing with exited states of the ligand) mechanism. For a purely Ln-centred transition to have a transition moment, the transition must contain in part some 5d orbital component to be parity allowed. This is mediated by the dissymmetric ligand allowing this mixing. Due to sum rules the integral of the whole band is hence zero.^46^ On the other hand, a dynamic mechanism involves polarisation from the ligand which allows for transitions to be slightly allowed. However, due to mixing with the ligand, the integral of the transition can be non-zero.^46^ The integral of the CD spectra of ΔYb/ΛYb show clear symmetric divergence from zero (Fig. S13), suggesting that some of the intensity of the CD spectra originates from polarisation from the ligand. Hence, designing ligands which facilitate mixing of the lanthanide orbitals with exited states of the ligand could provide a path towards very sensitive electric field coupling.

**Fig. 4:**
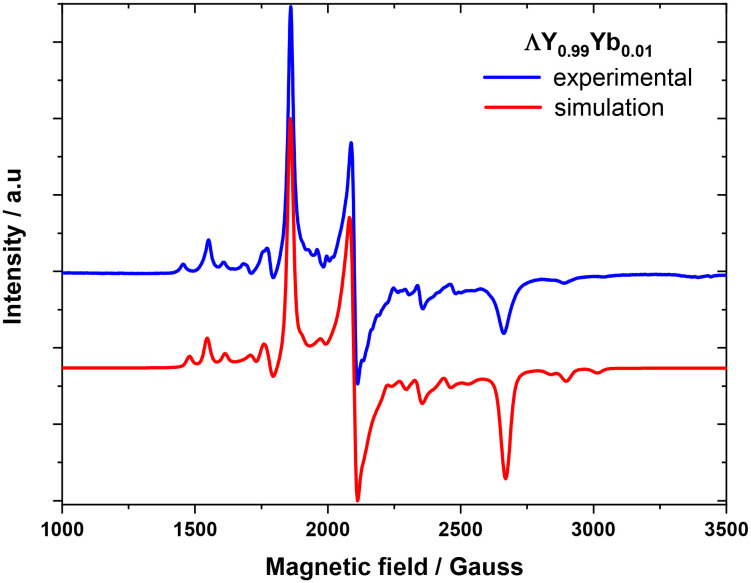
X-band (9.63 GHz) c.w.-E.P.R. of polycrystalline **ΛY**_**0.99**_**Yb**_**0.01**_, at 20 K.

Having shown that the Yb^III^ centre experiences a chiral environment due to the dissymmetry induced by the chiral ligand, we now examine whether condensation with a chiral amine perturbs its susceptibility to coherent manipulation by microwave pulses, as compared to LYb. Detailed information about the *g*- and hyperfine coupling tensor, *A*, of ΔYb/ΛYb was obtained from c.w.-EPR spectra on polycrystalline **ΛY**_**0.99**_**Yb**_**0.01**_ (Fig. 4). The c.w.-EPR spectrum was modelled as originating from an effective *S* = 1/2 system, due to the large zero field splitting of the eigenstates of the ^2^F_7/2_ term, resulting in a thermally isolated ground Kramers doublet. The following Hamiltonian was used to model the obtained c.w.-EPR spectra:
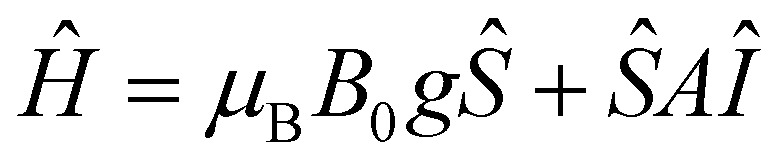


The fit (*χ*^2^ = 0.0163) matches the experimental data very well (Fig. 4) and results to the following best-fit parameters: *g*_*x*_ = 2.574(2), *g*_*y*_ = 3.274(0), *g*_*z*_ = 3.697(2), *A*_*x*_ = 523(4) MHz, *A*_*y*_ = 664(2) MHz, *A*_*z*_ = 766(4) MHz.

The resulting parameters are within the range observed for other similar systems based on the Yb(trensal) motive.^12,36,47^ In contrast to these other systems, ΔYb/ΛYb display rhombic *g*- and hyperfine exchange tensors, even though to a good approximation the molecule possesses trigonal symmetry. However, unlike similar molecules, ΔYb/ΛYb do not possess strict crystallographic trigonal symmetry, hence allowing the *g*- and hyperfine exchange tensors to be rhombic. These observations illustrate the importance of, and the necessity for, strict crystallographic symmetry for obtaining systems characterised by Hamiltonians of accordingly high symmetry.

**Fig. 5:**
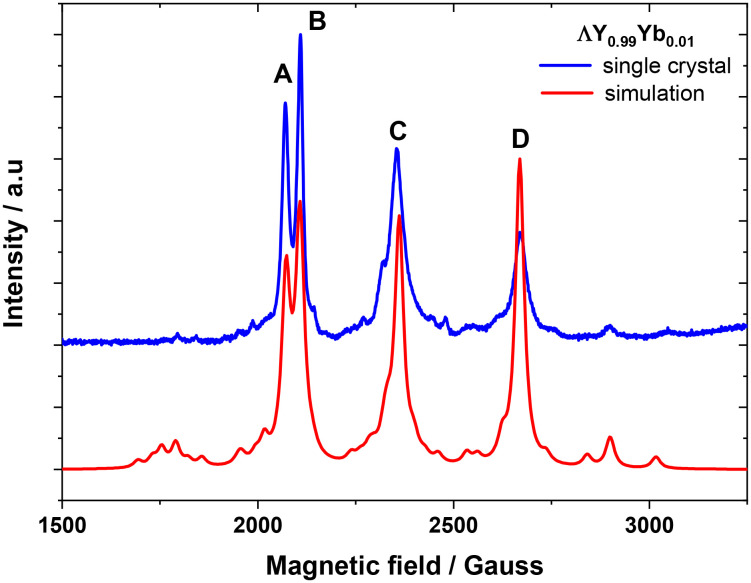
Single crystal EDFS of **ΛY**_**0.99**_**Yb**_**0.01**_ measured at 5 K using a standard Hahn echo sequence, as described in the experimental section. Main transitions are labelled **A–D**. Simulation (red) based on best fit from the polycrystalline sample.

**Fig. 6:**
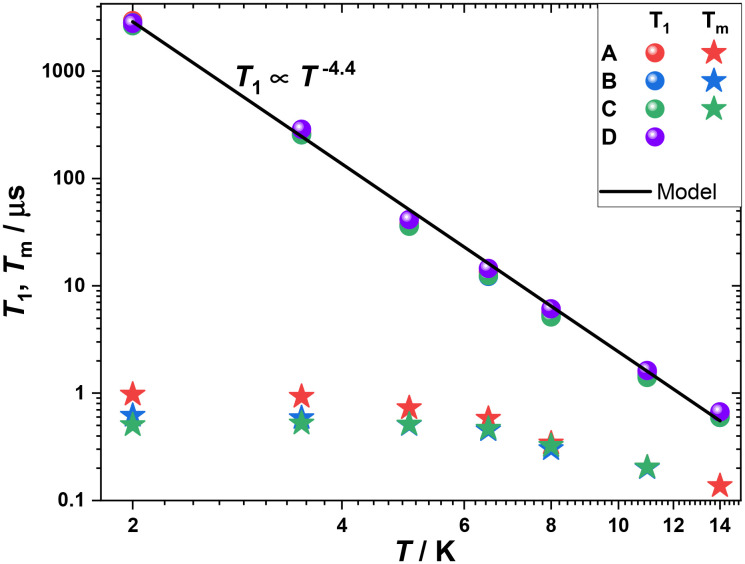
Temperature dependence of *T*_1_ and *T*_m_ for the four main transitions **A–D** of **ΛY**_**0.99**_**Yb**_**0.01**_. A model of *T*_1_ ∝ *T*^−4.4^ is shown as a black line.

Pulse-EPR measurements were conducted on a single crystal of **ΛY**_**0.99**_**Yb**_**0.01**_ for which orientation selectivity between the four different magnetically inequivalent sites of the unit cell was possible without overlapping contributions, as would be the case for a polycrystalline sample. This also results to long Rabi nutation traces possible due to the larger *B*_1_-field homogeneity over the single crystal, as compared to the one for polycrystalline powder samples which are intrinsically less homogeneous. The specific orientation was chosen such that a large magnetic field splitting between the observed lines was obtained (Fig. 5). The EDFS spectrum reveals 4 major lines, labelled **A–D** in Fig. 5, each corresponding to one of the 4 different magnetically inequivalent orientations of crystallographically identical Yb^III^ sites within the crystal possessing no nuclear spin (*I* = 0). The smaller peaks observed originate from hyperfine coupling to the less abundant ^173^Yb and ^171^Yb isotopes possessing a nuclear spin, *I* = 5/2 and *I* = 1/2, respectively. The dynamics of the **A–D** lines (Fig. S23–S30 and Tables S7 and S8) originating from *I* = 0 isotopes were measured for all 4 inequivalent orientations within the sample (Fig. 5 and 6). Slightly longer *T*_m_'s than for Yb(trensal) were observed at similar doping level (1%), likely due to the methyl-benzyl arms increasing the average distance between paramagnetic centres. *T*_1_ shows a steep temperature dependence, *T*_1_ ∝ *T*^−4.4^, which is also observed for similar ytterbium compounds due to the large orbital angular momentum of the ground state resulting in strong coupling to the lattice. Consequently, *T*_m_ slowly decreases with increasing temperatures, with 14 K being the highest temperature at which an echo is detectable, due to *T*_1_ limiting *T*_m_ at higher temperatures. This limit is however slightly lower than for Yb(trensal) which is *T*_1_-limited at around 20 K. This difference could originate from the increase of the number of phonons resulting from the introduction of the extra benzyl amine moieties.

**Fig. 7:**
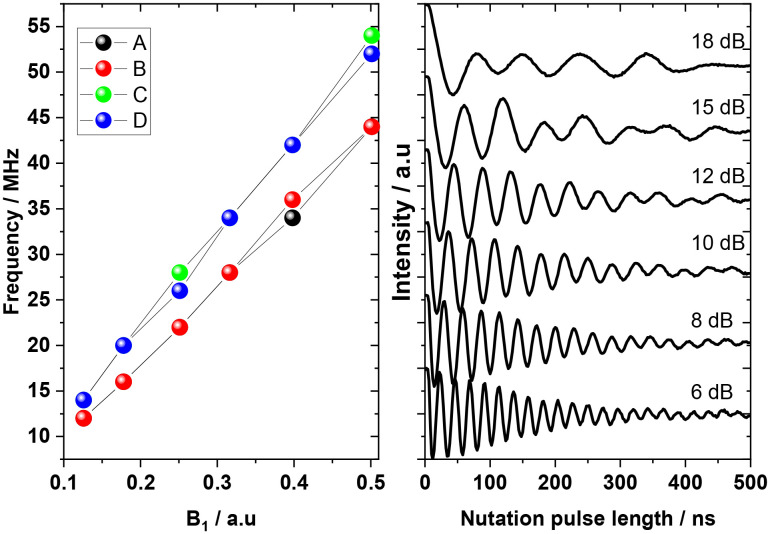
Left: Rabi frequencies for the four transitions **A–D** of **ΛY**_**0.99**_**Yb**_**0.01**_*versus B*_1_. Right: Rabi nutation at various power levels for transition **A**.

Transient nutation experiments display clear oscillations of the echo intensity (Rabi oscillations) with nutation pulse duration (Fig. 7 and S31–S34). As expected, the Rabi frequencies, extracted by Fourier Transform of transient nutation traces (Fig. S35–S38), show a linear dependence to microwave power (*B*_1_). The Rabi frequencies at a given *B*_1_ come in pairs with the **A** and **B** resonances in general showing slower nutation frequencies than the **C** and **D** ones, due to the anisotropic *g*-tensor. This indicates that the orientation of the sample plays an important role for the Rabi frequency, for systems with large *g*-tensor anisotropy. Transitions **A–D** display more than 30 coherent oscillations indicative of a homogenous *B*_1_ field across the sample. Observation of a large number of Rabi oscillations is indicative of potential for high fidelity of operations to be implemented on the system.

## Conclusion

We show herein that post functionalization of an aldehyde containing lanthanide complex (LYb) with chiral primary amines provides a route to chiral lanthanide complexes. Circular dichroism and absorption spectroscopy studies of the obtained chiral complexes evidenced clear f–f transitions for Yb characterised by large *g*_abs_ values of the order of 0.07 for which a dynamic mechanism involving the ligand contributes to the CD intensity. This provides a potential design criterion for the development of molecular materials exhibiting strong magnetoelectric coupling. Furthermore, we show that under the conditions of this study ΔYb/ΛYb display a phase memory time of about 0.5 to 1 μs and that the electronic spin can be coherently manipulated by microwave pulses. The frequency of the observed Rabi oscillations is orientation dependent for these systems characterised by strongly anisotropic *g*-factor. Hence, we create herein an Yb(trensal) analogue showing chiroptical properties while retaining similar coherent dynamic properties as the parent LYb. These results establish the family of Yb(trensal) complexes and derivatives as an avenue for further studies of chirality induced effects on the coherent dynamics of molecular materials relevant for quantum technologies. The complexes discussed herein being enantiopure, offer the advantage that they might give access to studies that might not be feasible in crystals of chiral molecular complexes containing both enantiomers, as is the case for example for Yb(trensal).

## Author contributions

The project was jointly conceived by CDB, SHH and SP. CDB and SHH prepared the samples. BEB and SHH performed the measurements. SHH performed the data analysis, supervised by SP and BEB. The manuscript was written jointly by all authors, who all have read and agreed to the final version of the manuscript.

## Conflicts of interest

There are no conflicts to declare.

## Supplementary Material

QI-012-D5QI00977D-s001

QI-012-D5QI00977D-s002

## Data Availability

The research data supporting this publication will be freely accessible at https://doi.org/10.17630/443f0aa3-c7a6-4b8c-9abe-874d88c5b6f3. Supplementary information is available. See DOI: https://doi.org/10.1039/d5qi00977d. CCDC 2428735 (ΔYb), 2428867 (ΛYb), 2428550 (ΔY) and 2428518 (ΛY) contains the supplementary crystallographic data for this paper.^48*a*–*d*^ All structures were measured at 120 K.
