# Supplementary material for: Chiral molecular 4f qubits by post functionalization
Source: Inorg Chem Front. 2025 Sep 1;12(24):8000–7. doi: 10.1039/d5qi00977d (PMC12401168; doi:10.1039/d5qi00977d)
Supplement: QI-012-D5QI00977D-s001 [file QI-012-D5QI00977D-s001.pdf]

Supplementary Information (SI) for Inorganic Chemistry Frontiers.
This journal is © the Partner Organisations 2025

Supporting information for
Chiral molecular 4f qubit by post functionalization

Steen H. Hansen, Christian D. Buch, Bela E. Bode and Stergios Piligkos

[a]Department of Chemistry, University of Copenhagen, Universitetsparken 5, Denmark

[b]EaStCHEM School of Chemistry, Biomedical Sciences Research Complex, and Centre of Magnetic Resonance, University of St Andrews, North Haugh, St Andrews KY16 9ST, UK

Contents

Elemental Analysis 2

ICP-MS 2

Crystallography 3

IR 4

Circular dichroism 17

Powder X-ray diffraction 18

^{1}H-NMR 19

MALDI-MS 22

Pulse EPR 26

T_1 time traces 26

T_m time traces 30

Rabi nutations 34

EasySpin script for simulation of the Single Crystal EDFS 40

Elemental Analysis

Table S1. Elemental analysis of **ΔYb** and **ΛYb for chemical formula $C_{57}H_{60}N_7O_3Yb$**.

	% C found (calculated)	% H found (calculated)	% N found (calculated)
**ΛYb**	64.2 (64.3)	5.8 (5.7)	9.2 (9.2)
**ΔYb**	64.1 (64.3)	5.67 (5.7)	9.1 (9.2)

ICP-MS

6.05 mg of nominally Λ-$Yb_{0.01}Y_{0.99}$ were digested in 5.0 mL trace analysis nitric acid and diluted to 50 mL. 5 mL of this solution was taken and diluted to 20 mL, affording a final concentration of 6.05 mg / 200 mL, corresponding to an expected 53.4 ppb for Yb.

The diluted Λ-$Yb_{0.01}Y_{0.99}$ solution was further diluted twice at 5:50 mL to afford an expected 27.1 ppb solution for Y.

Found results: Yb: 53.0987 ppb and Y: 20.3380 ppb, corresponding to doping percent of 1.32% and a composition of Λ-$Yb_{0.013}Y_{0.987}$.

Crystallography

Table S2. Crystallographic data of **ΔYb** and **ΛYb**

	**ΛYb**	**ΔYb**
Formula	$C_{57}H_{60}N_7O_3Yb$	
Molar mass / g mol^{-1}	1064.20	
Temprerature / K	120	
Crystal system	Orthorhombic	
Space groupe	*P*212121	
a / Å	12.6436	12.6592
b / Å	19.7460	19.7428
c / Å	20.9552	20.9378
V/ Å^3	5231.7	5232.9
Z	4	
Density /g cm-^{3}	1.351	1.347
μ	1.837	
F_{000}	2180	2168
Radiation	Mo Kα (λ=0.71073 Å)	
2θ –range / deg	4.4-58.256	3.822 to 55.757
Reflections collected	74587	54498
Independent reflections	14033	12452
R_{int}, R_σ	0.0352, 0.0352	0.0960, 0.0576
Parameters/restrictions	619/0	619/0
Goodnes of fit on F^2	1.071	1.049
*R*1, w*R*2, *I*>2a	0.0219, 0.0503	0.0326, 0.0712
*R*1, w*R*2, *I*>2σ, All Data	0.0254, 0.0516	0.0443, 0.0766
Larges diff. peak/hole /eÅ-^{3}	0.88, -0.57	1.39, -1.13
Flack parameter	-0.013(2)	-0.011(5)
	**ΛY**	**ΔY**
Formula	$C_{57}H_{60}N_7O_3Y$	
Molar mass / g mol^{-1}	981.06	
Temprerature / K	120	
Crystal system	Orthorhombic	
Space groupe	*P*212121	
a / Å	12.5967	12.5882
b / Å	19.7174	19.6767
c / Å	21.2454	21.2236
V/ Å^3	5276.8	5257
Z	4	4
Density /g cm-^{3}	1.2336	1.238
μ	1.166	1.159
F_{000}	2044.7	2056
Radiation	Mo Kα (λ=0.71073 Å)	
2θ –range / deg	3.84 to 46.52	4.14 to 51.362
Reflections collected	36670	46974
Independent reflections	7525	9881
R_{int}, R_σ	0.0861, 0.0756	0.0585, 0.0634
Parameters/restrictions	619/0	619/0
Goodnes of fit on F^2	0.986	1.009
*R*1, w*R*2, *I*>2σ	0.0353, 0.0641	0.0314, 0.0654
*R*1, w*R*2, *I*>2a, All Data	0.0525, 0.0712	0.0447, 0.0698
Larges diff. peak/hole /eÅ-^{3}	0.35, -0.33	0.37,-0.30
Flack parameters	-0.015(4)	-0.010(3)

Table S3. Selected bond-lengths for **ΛYb**

Bond	Bond length/ Å
Yb01 – N006	2.599(3)
Yb01 – N00A	2.414(2)
Yb01 – N005	2.398(2)
Yb01 – N007	2.433(3)
Yb01 – O002	2.158(2)
Yb01 – O003	2.155(2)
Yb01 – O004	2.1532(19)

Table S4. Selected bond-lengths for **ΔYb**

Bond	Bond length/ Å
Yb1 – N2	2.595(4)
Yb1 – N1	2.408(4)
Yb1 – N5	2.430(5)
Yb1 – N7	2.395(4)
Yb1 – O1	2.159(3)
Yb1 – O3	2.155(3)
Yb1 – O5	2.156(4)

Table S5. Selected bond-lengths for **ΛY**

Bond	Bond length/ Å
Y001 – N00A	2.629(4)
Y001 – N005	2.452(4)
Y001 – N006	2.442(4)
Y001 – N009	2.479(5)
Y001 – O002	2.174(3)
Y001 – O003	2.180(3)
Y001 – O004	2.172(3)

Table S6. Selected bond-lengths for **ΔY**

Bond	Bond length/ Å
Y001 – N00B	2.623(3)
Y001 – N005	2.434(3)
Y001 – N006	2.454(3)
Y001 – N008	2.472(3)
Y001 – O004	2.175(2)
Y001 – O002	2.178(2)
Y001 – O003	2.175(2)

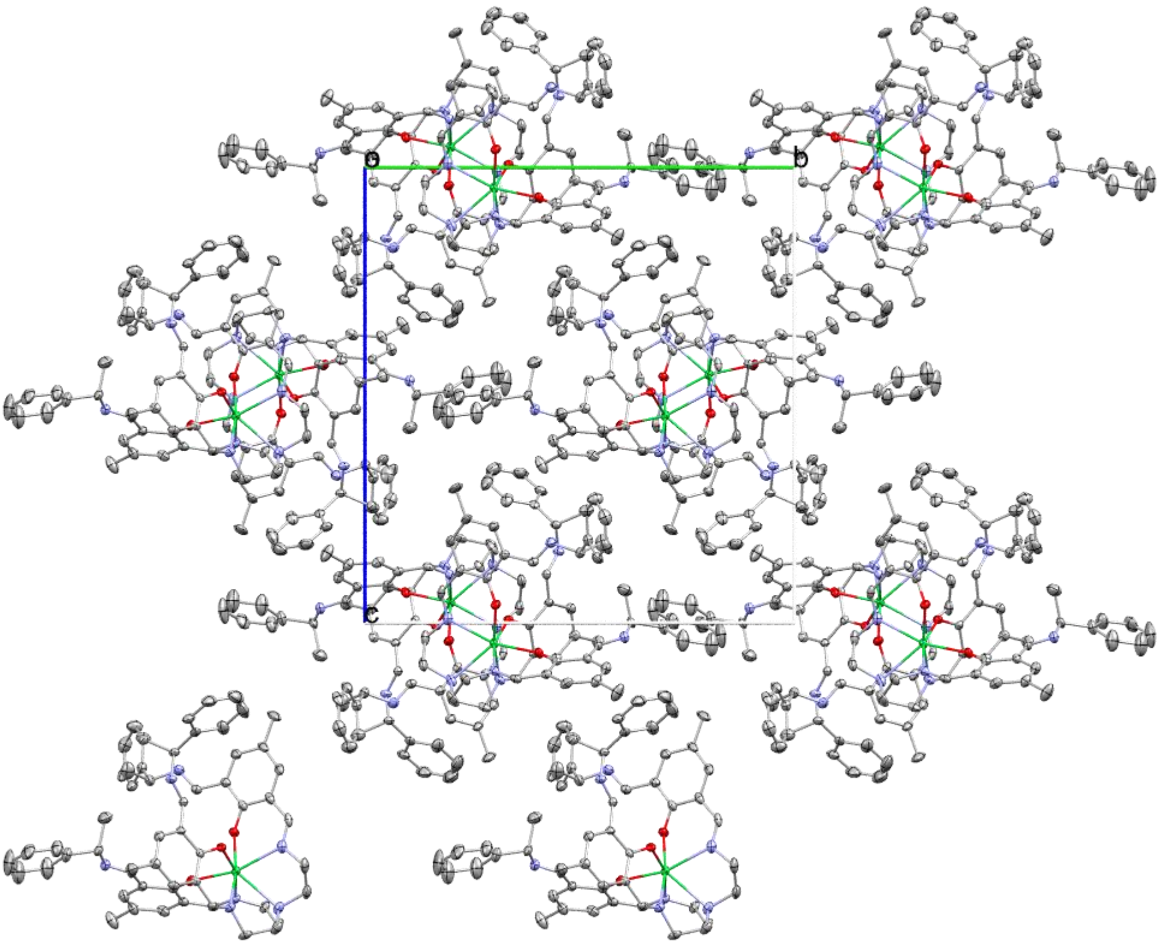

Figure S1. Crystal packing of **ΔYb** viewed along the a-axis.

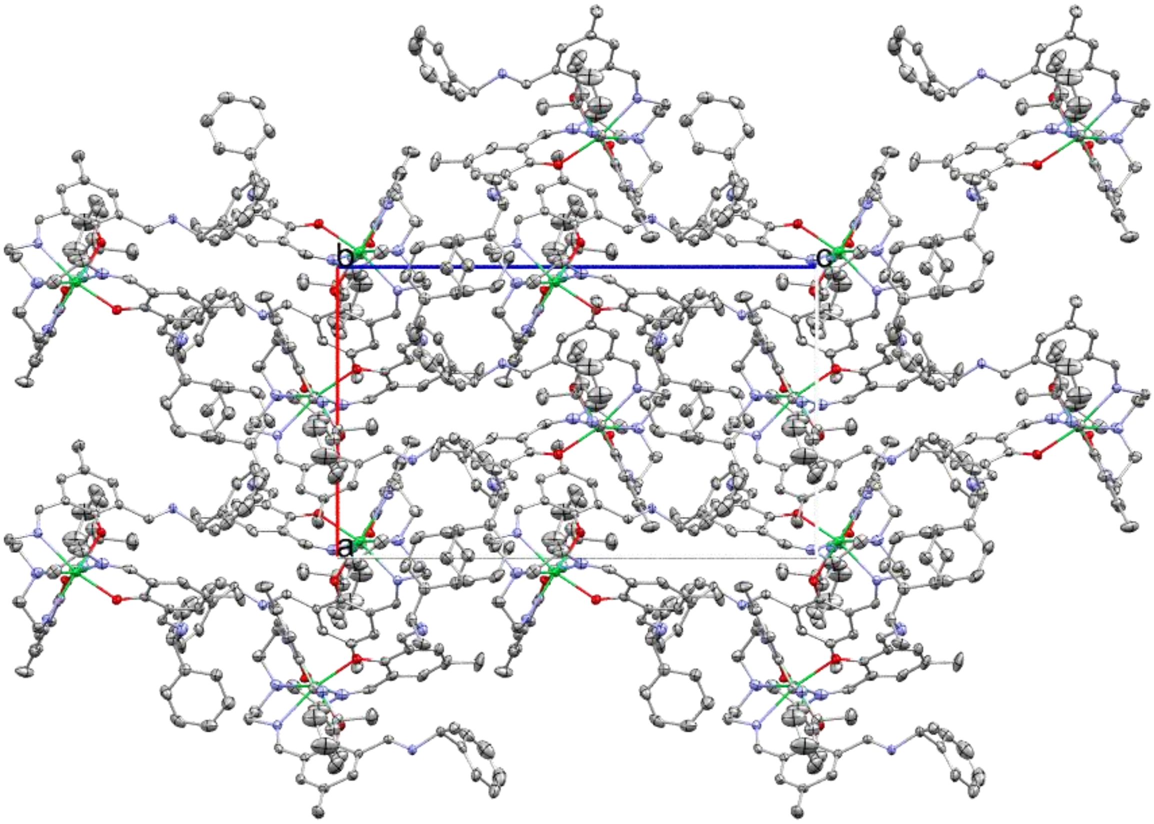

Figure S2. Crystal packing of **ΔYb** viewed along the b-axis.

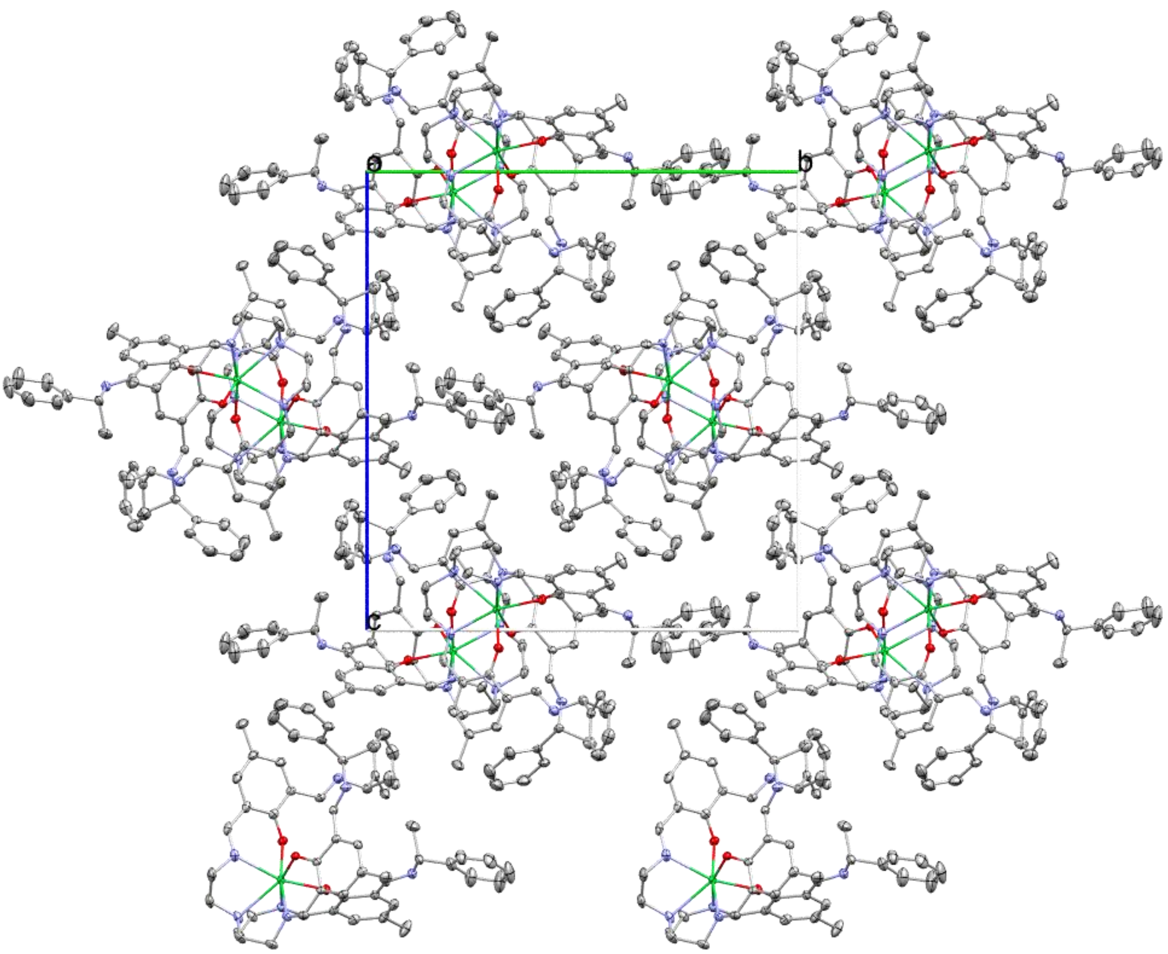

Figure S3. Crystal packing of **ΛYb** viewed along the a-axis.

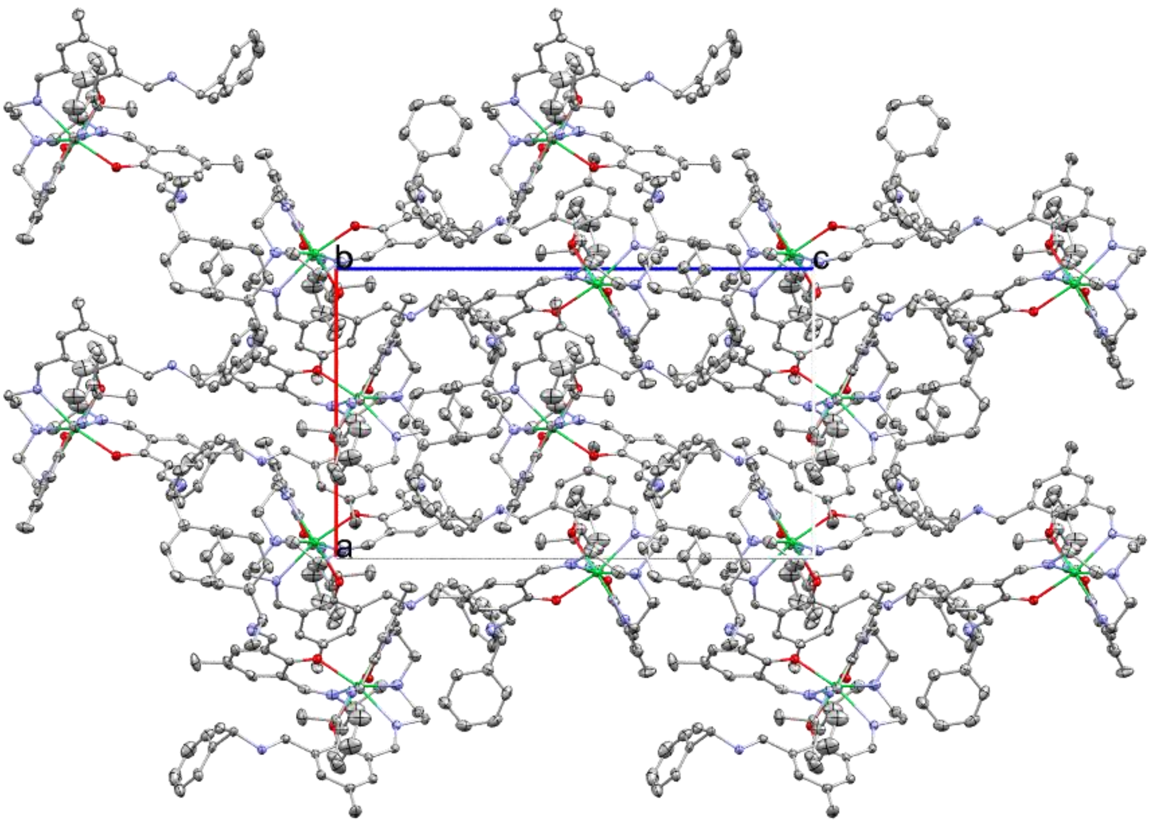

Figure S4. Crystal packing of **ΛYb** viewed along the b-axis.

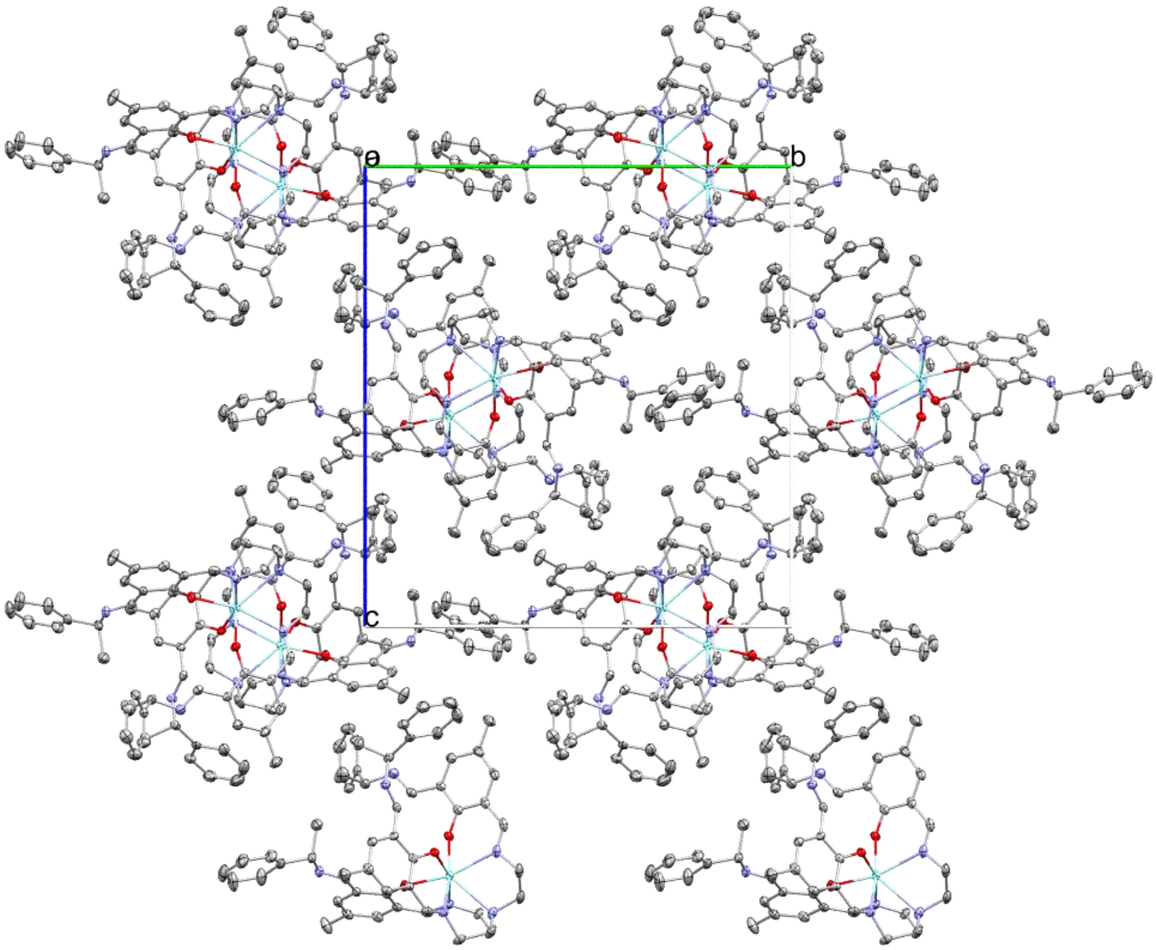

Figure S5. Crystal packing of **ΔY** viewed along the a-axis.

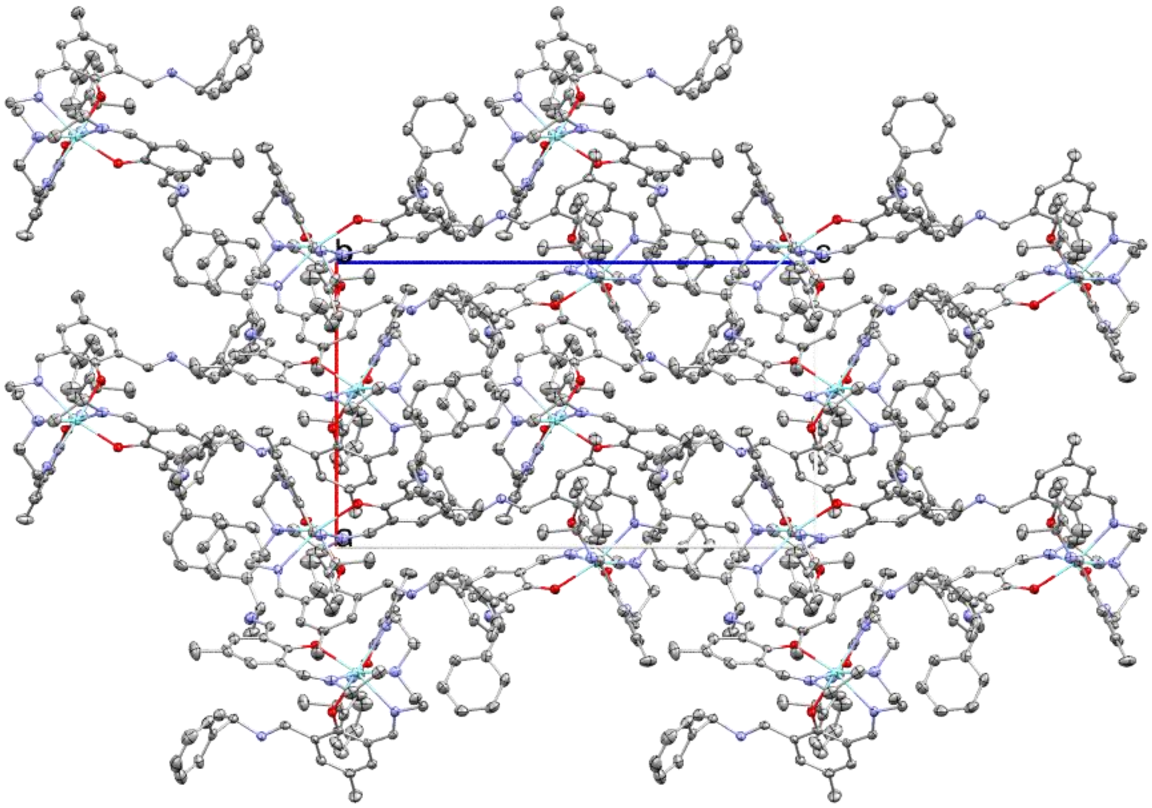

Figure S6. Crystal packing of **ΔY** viewed along the b-axis.

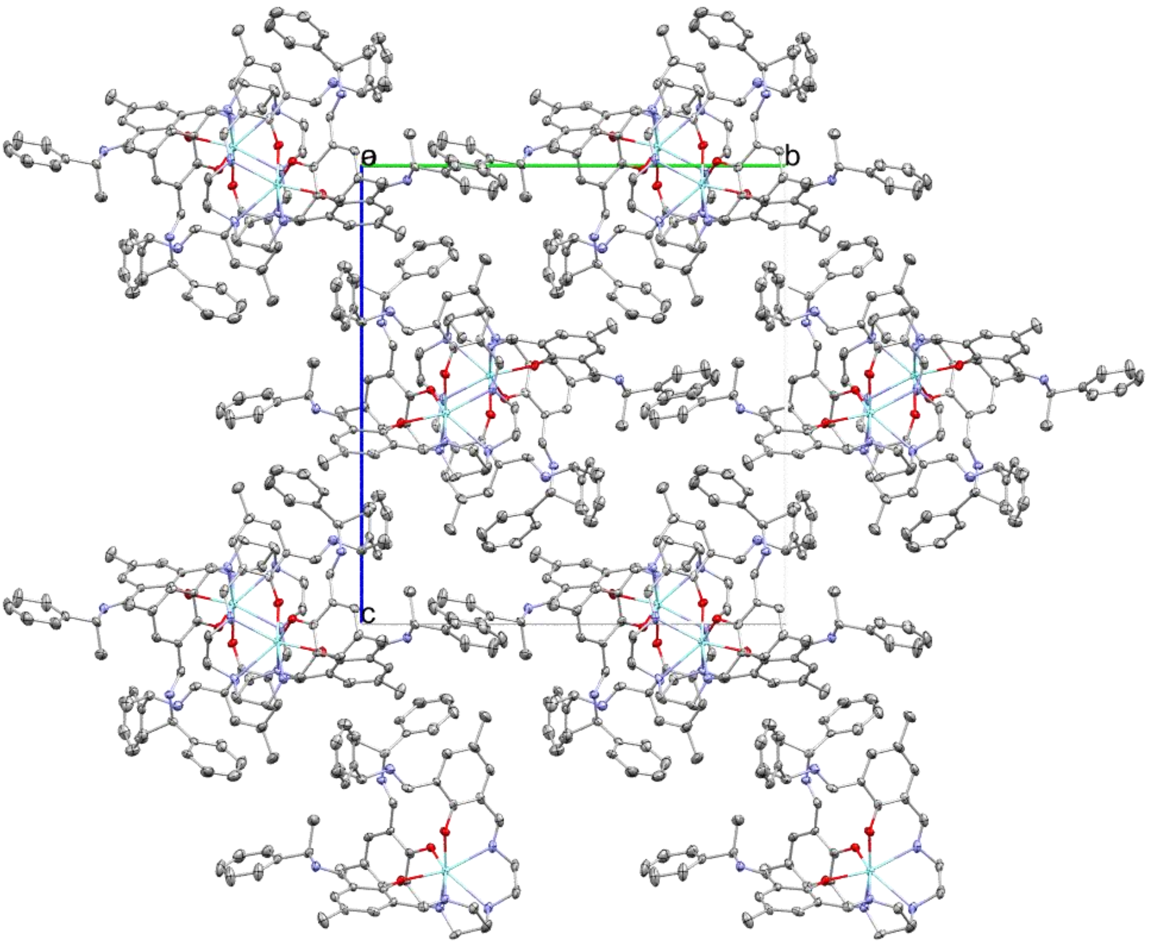

Figure S7. Crystal packing of **ΛY** viewed along the a-axis.

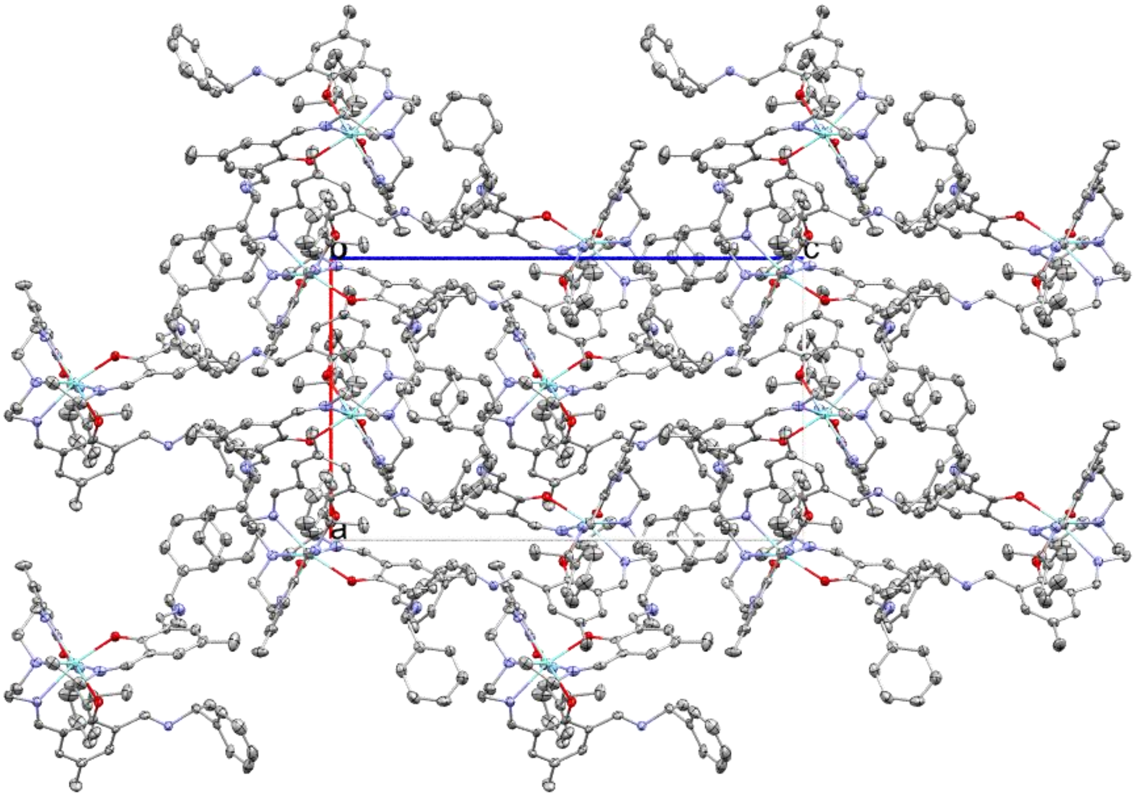

Figure S8. Crystal packing of **ΛY** viewed along the b-axis.

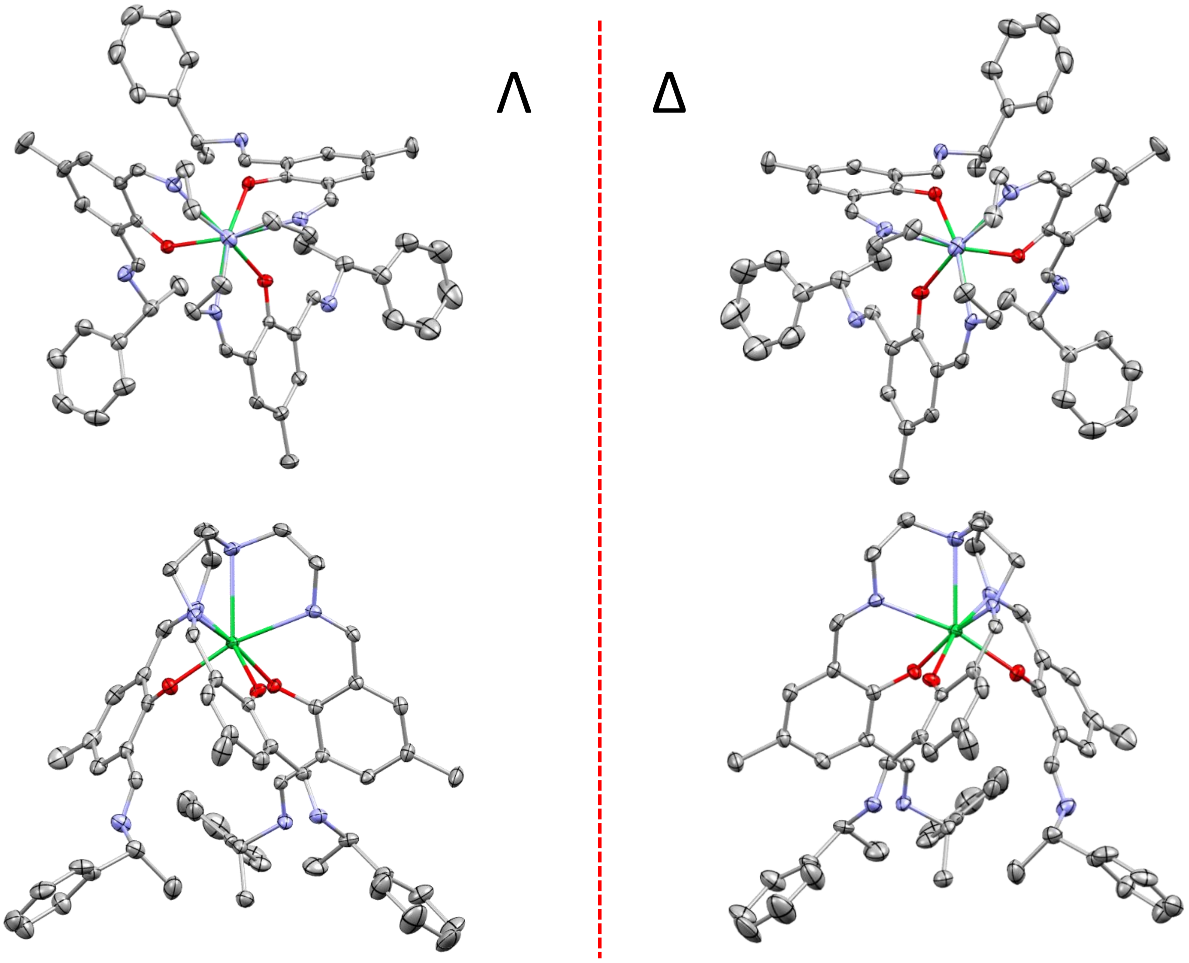

Figure S9. Top-down (top) and side (bottom) view of **ΛYb** (left) and **ΔYb** (right).

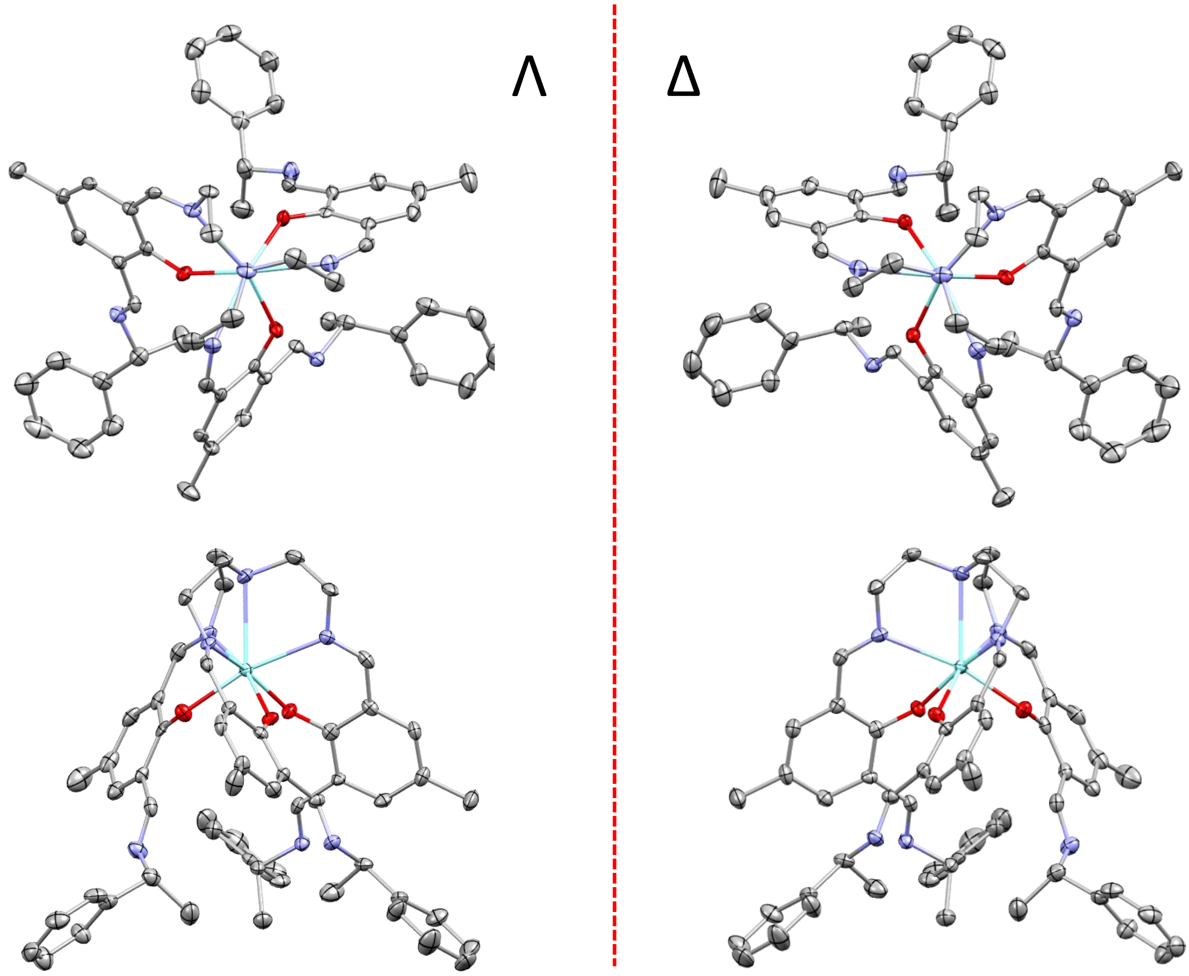

Figure S10. Top-down (top) and side (bottom) view of **ΛY** (left) and **ΔY** (right).

IR

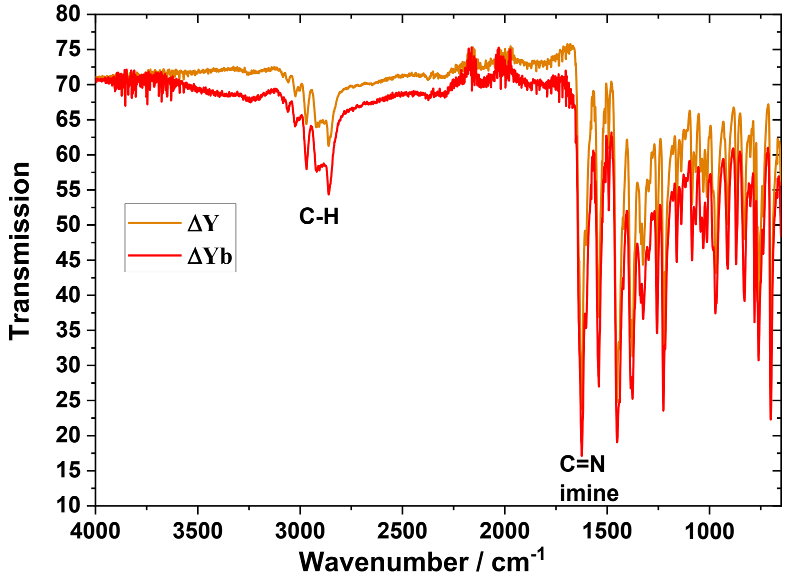

Figure S11. IR of **ΔYb** and **ΔY**, showing very similar IR spectra. In particular, they both show absence of carbonyl stretch frequencies, confirming that Schiff-base condensation to imines is complete.

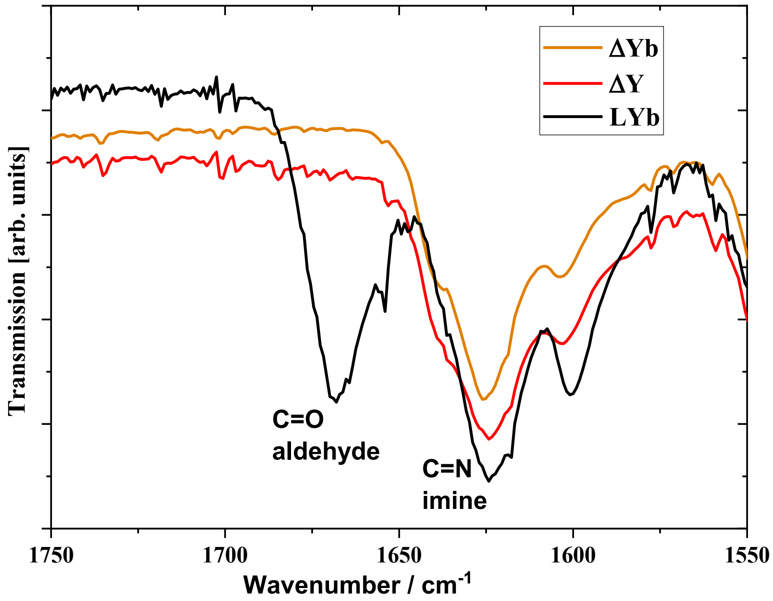

Figure S12. IR in the region of aldehyde and imine stretches for **ΔYb, ΔY** and **LYb**. Upon functionalization the aldehyde stretch above 1650 cm^{-1} disapears and only the imine stretch frequency persists.

Circular dichroism

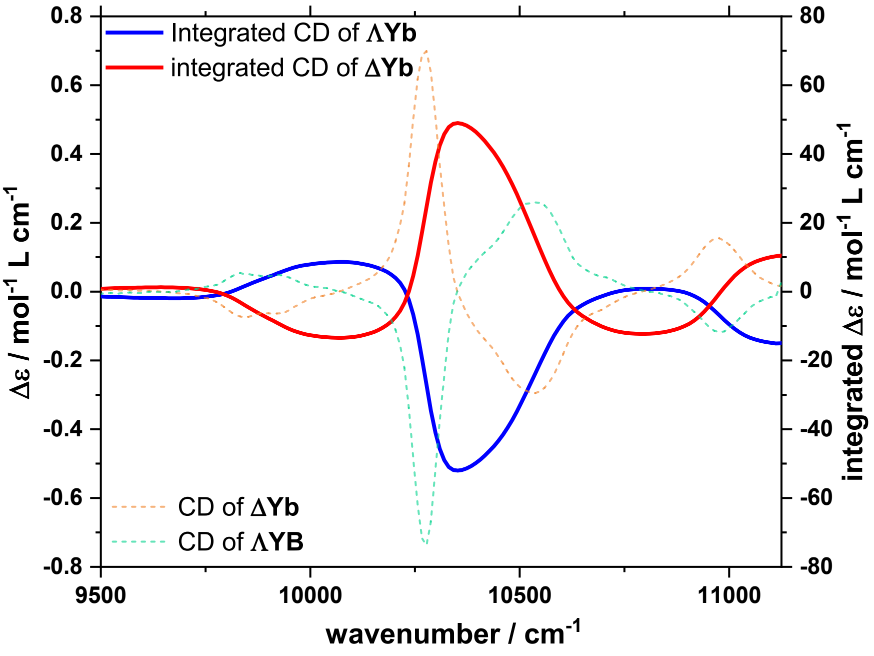

Figure S13. CD and integrated CD spectra of **ΔYb** and **ΛYb.**

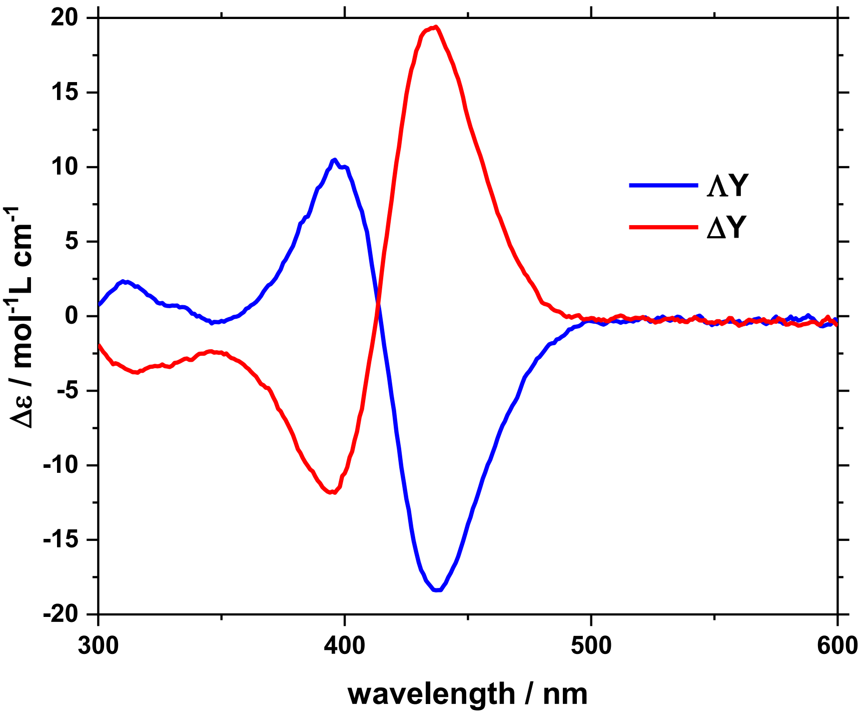

Figure S14. CD of **ΔYb** and **ΛYb** in the UV/Vis.

Powder X-ray diffraction

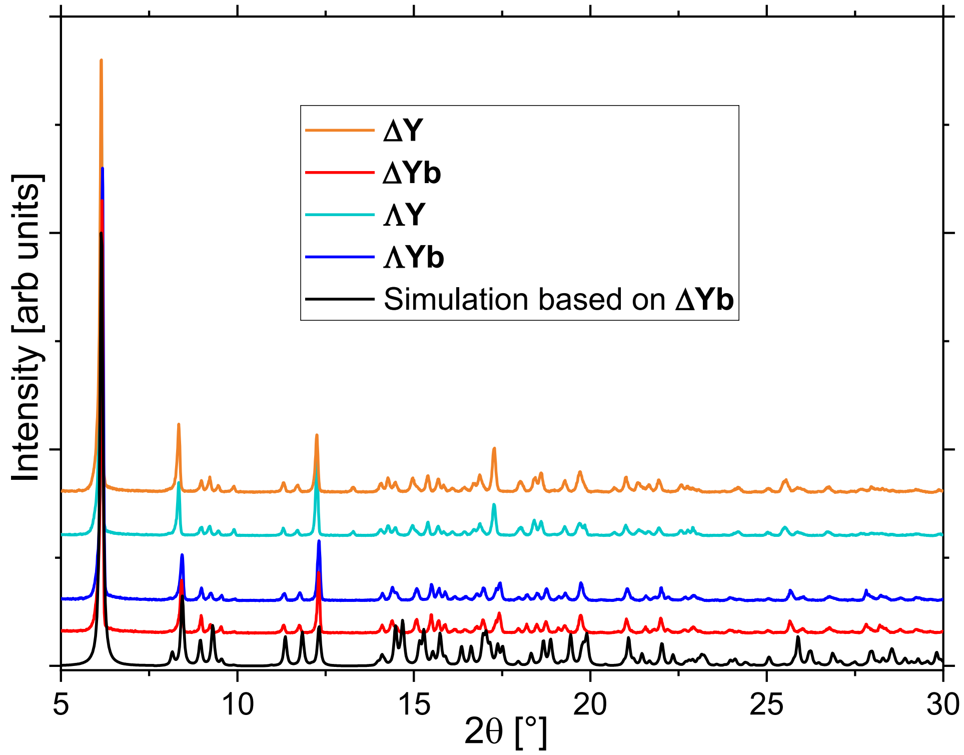

Figure S15. Powder X-ray diffraction of **ΔYb**/**ΛYb** and **ΔY**/**ΛY** compared to a simulation from a single crystal of **ΔYb**.

^{1}H-NMR

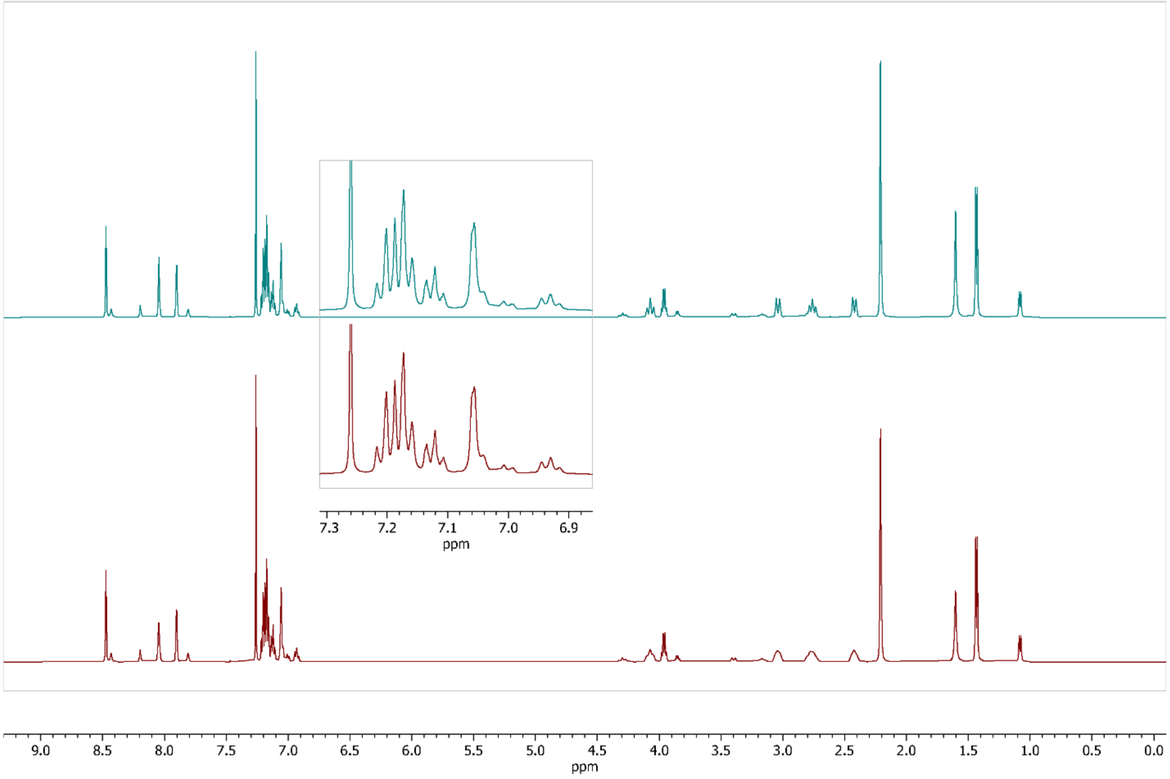

Figure S16. ^{1}H-NMR in $CDCl_3$. Top is **ΛY** and bottom is **ΔY**. The two spectra are virtually identical.

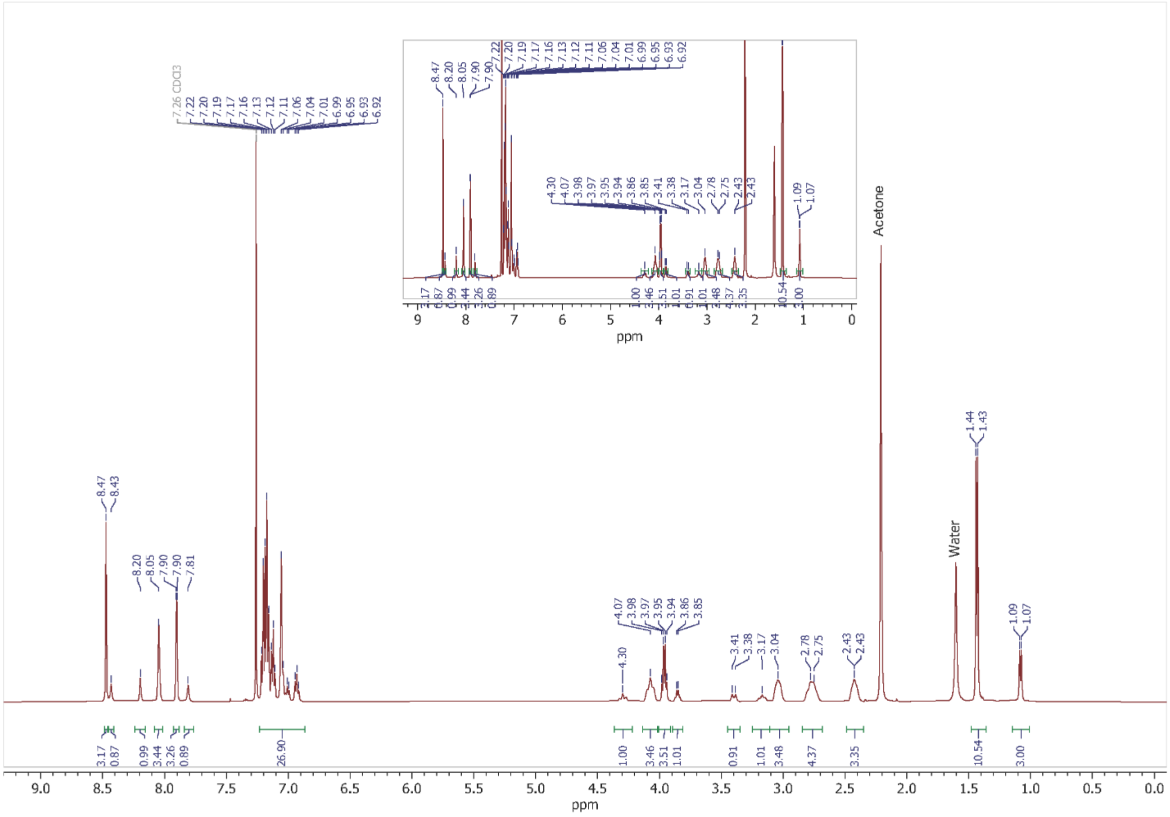

Figure S17. ¹H-NMR of **ΔY** in $CDCl_3$. More than 12 reonances and summed integrations near 60 suggest loss of C3 symmetry in solution at room temperature.

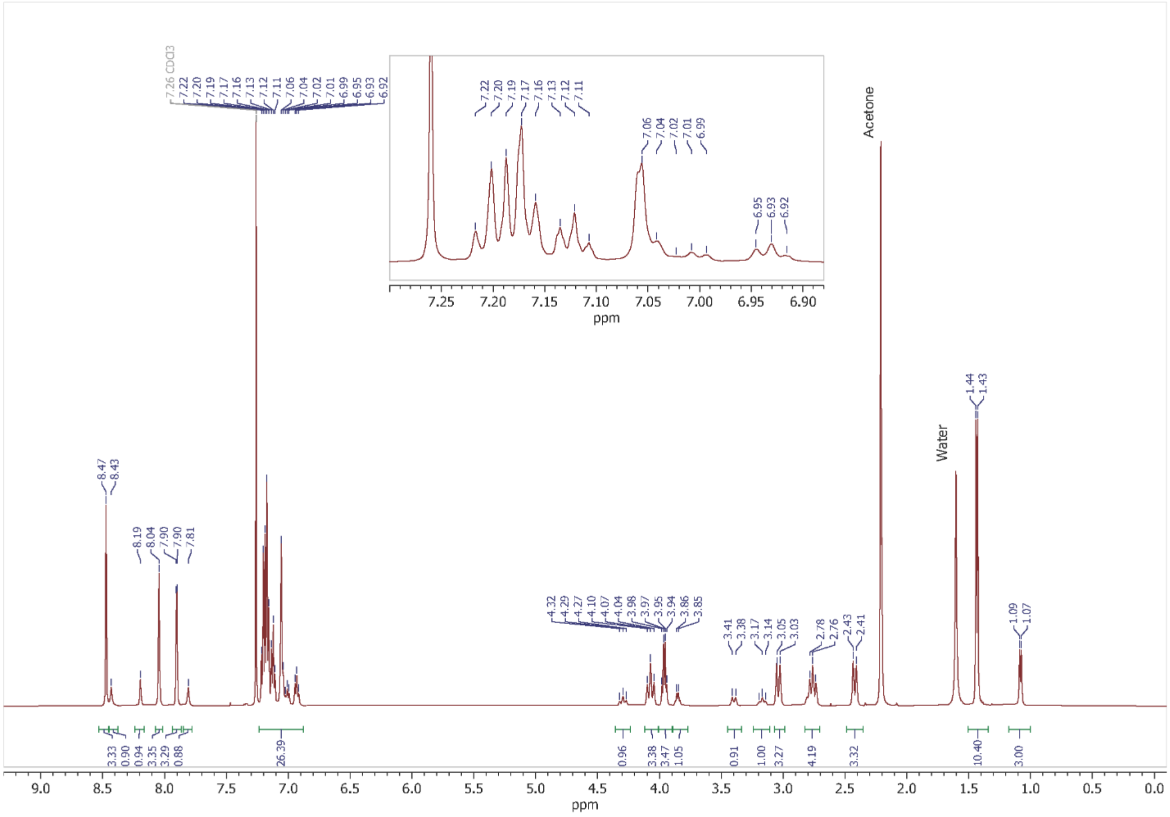

Figure S18. ^{1}H-NMR of **ΛY** in $CDCl_3$. More than 12 reonances and summed integrations near 60 suggest loss of C3 symmetry in solution at room temperature.

MALDI-MS

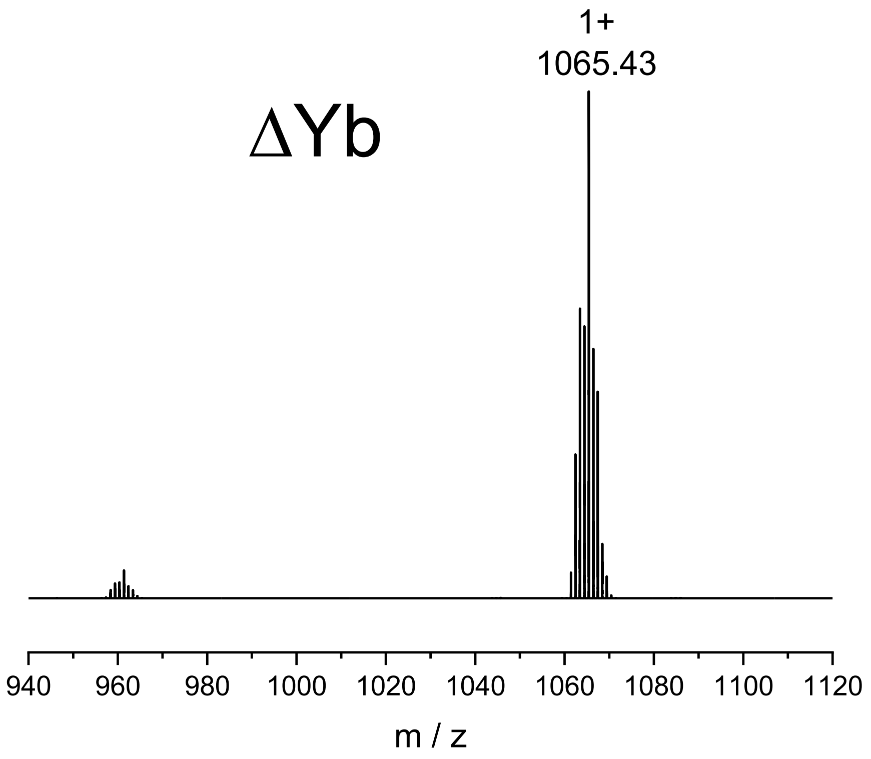

Figure S19. Positive ion mode MALDI-MS of **ΔYb.**

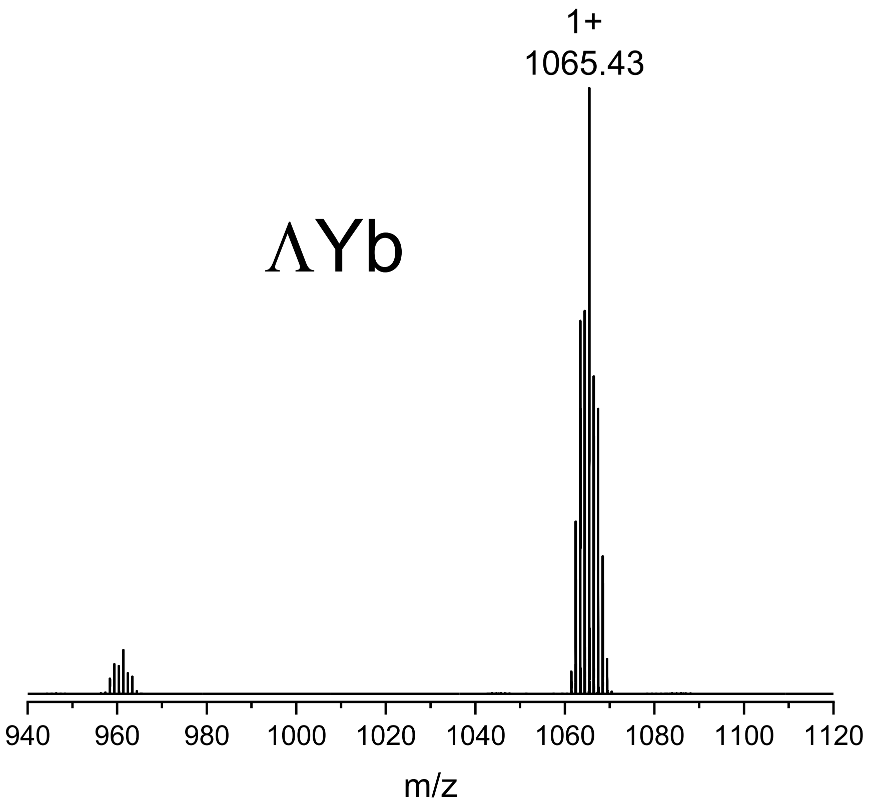

Figure S20. Positive ion mode MALDI-MS of **ΛYb.**

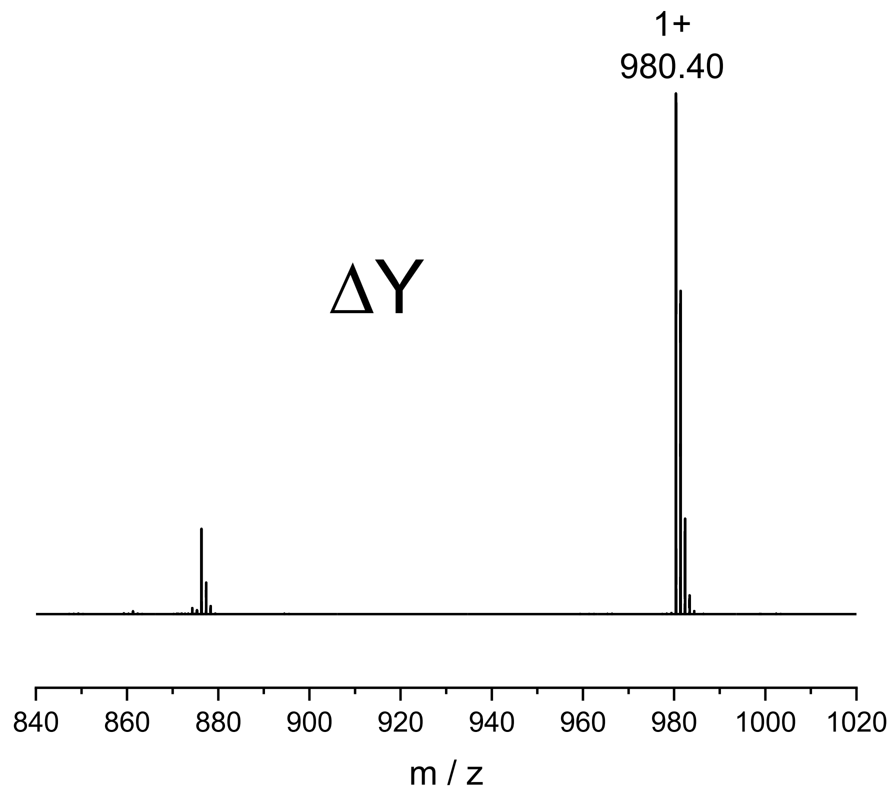

Figure S21. Positive ion mode MALDI-MS of **ΔY.**

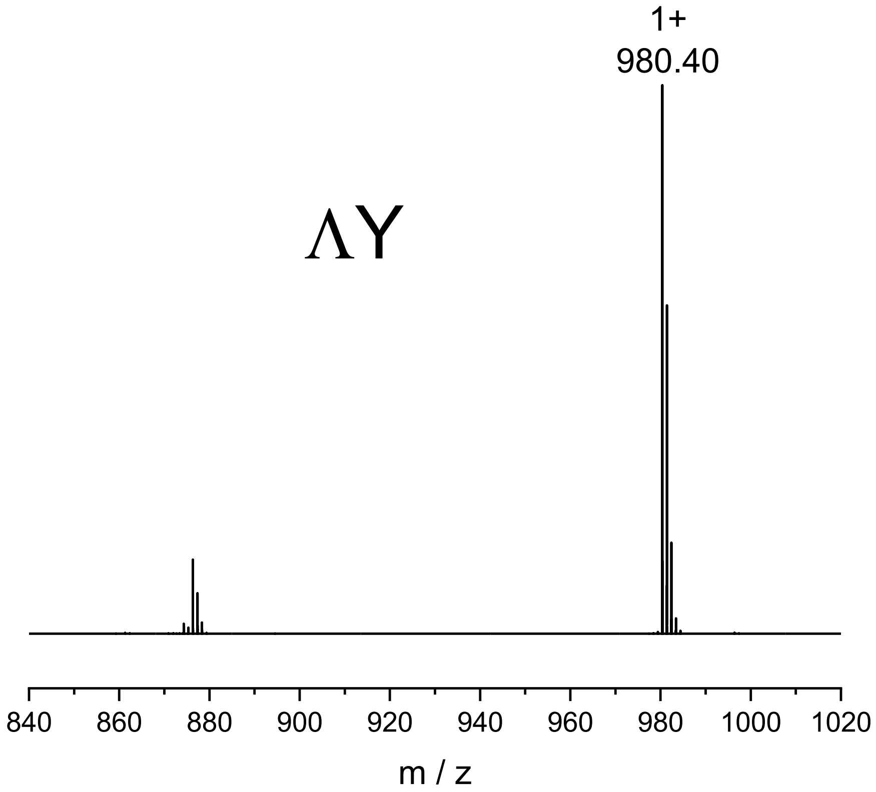

Figure S22. Positive ion mode MALDI-MS of **ΛY.**

Pulse EPR

T_1 time traces

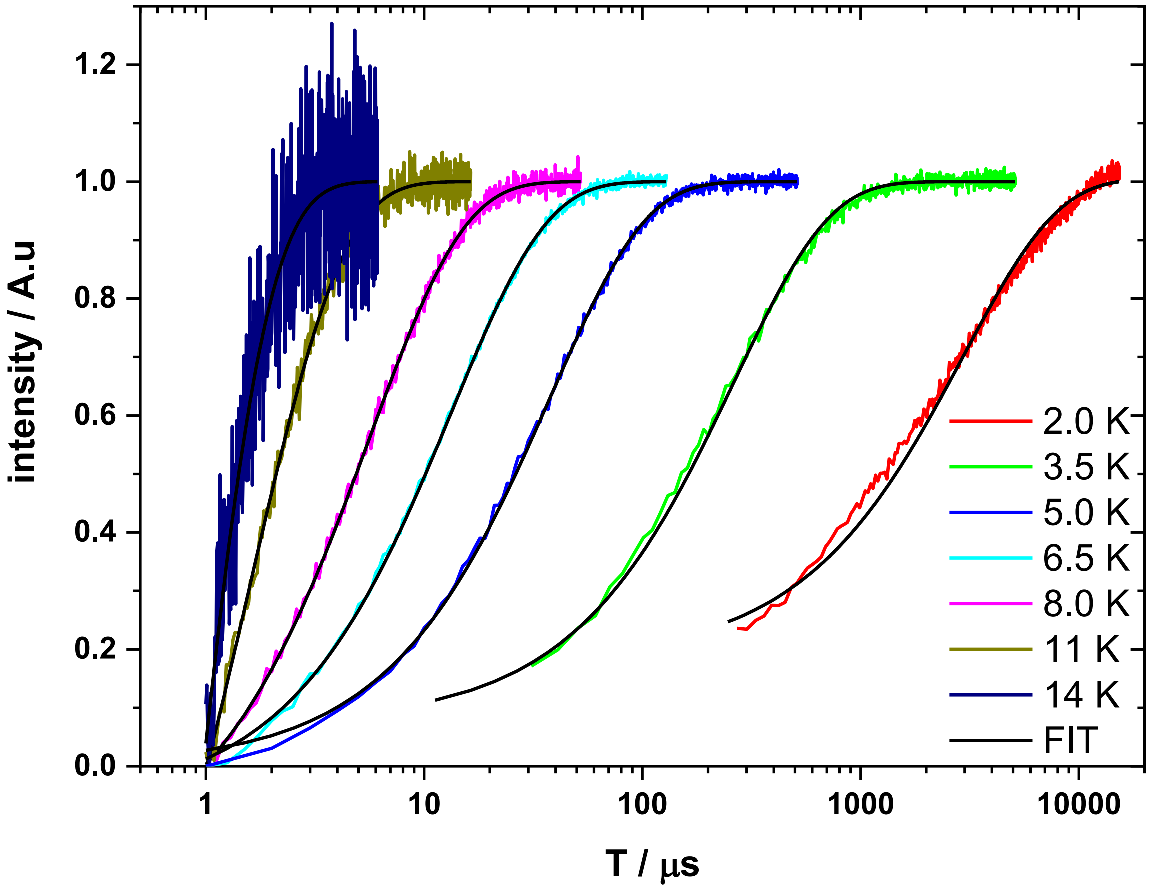

Figure S23. Inversion-recovery traces for transition **A** of **ΔYb** at 2070 Gauss. The experimental curves are vertically shifted and normalised to the equilibrium magnetisation at each temperature.

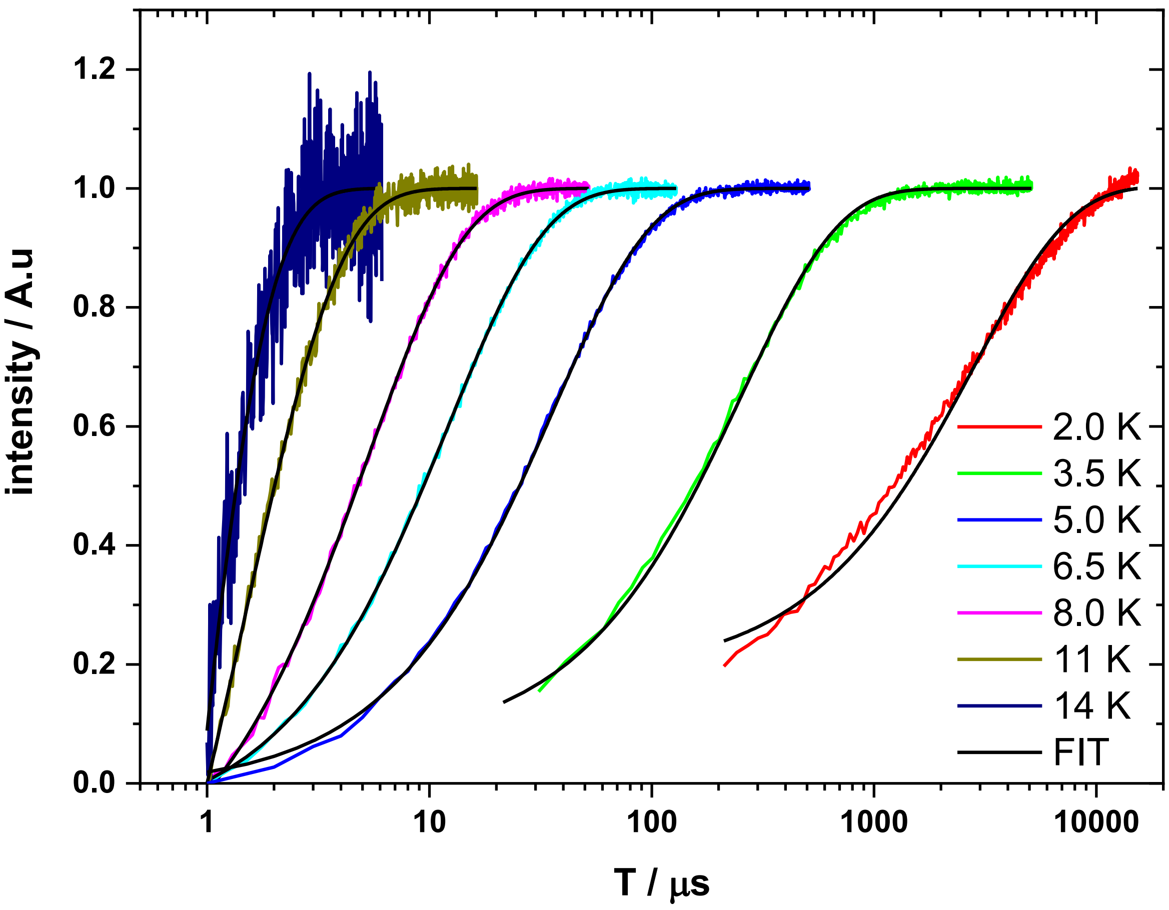

Figure S24. Inversion-recovery traces for transition **B** of **ΔYb** at 2109 Gauss. The experimental curves are vertically shifted and normalised to the equilibrium magnetisation at each temperature.

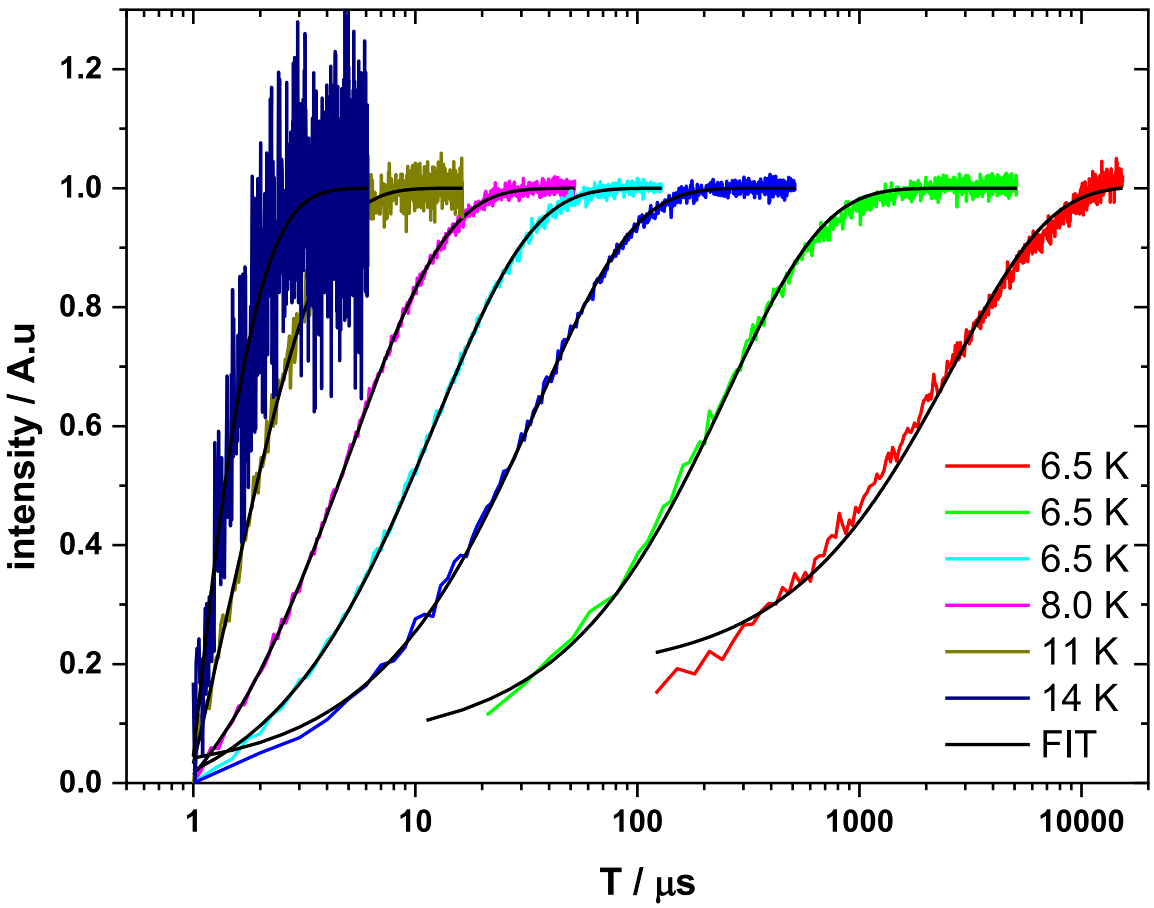

Figure S25. Inversion-recovery traces for transition **C** of **ΔYb** at 2356 Gauss. The experimental curves are vertically shifted and normalised to the equilibrium magnetisation at each temperature.

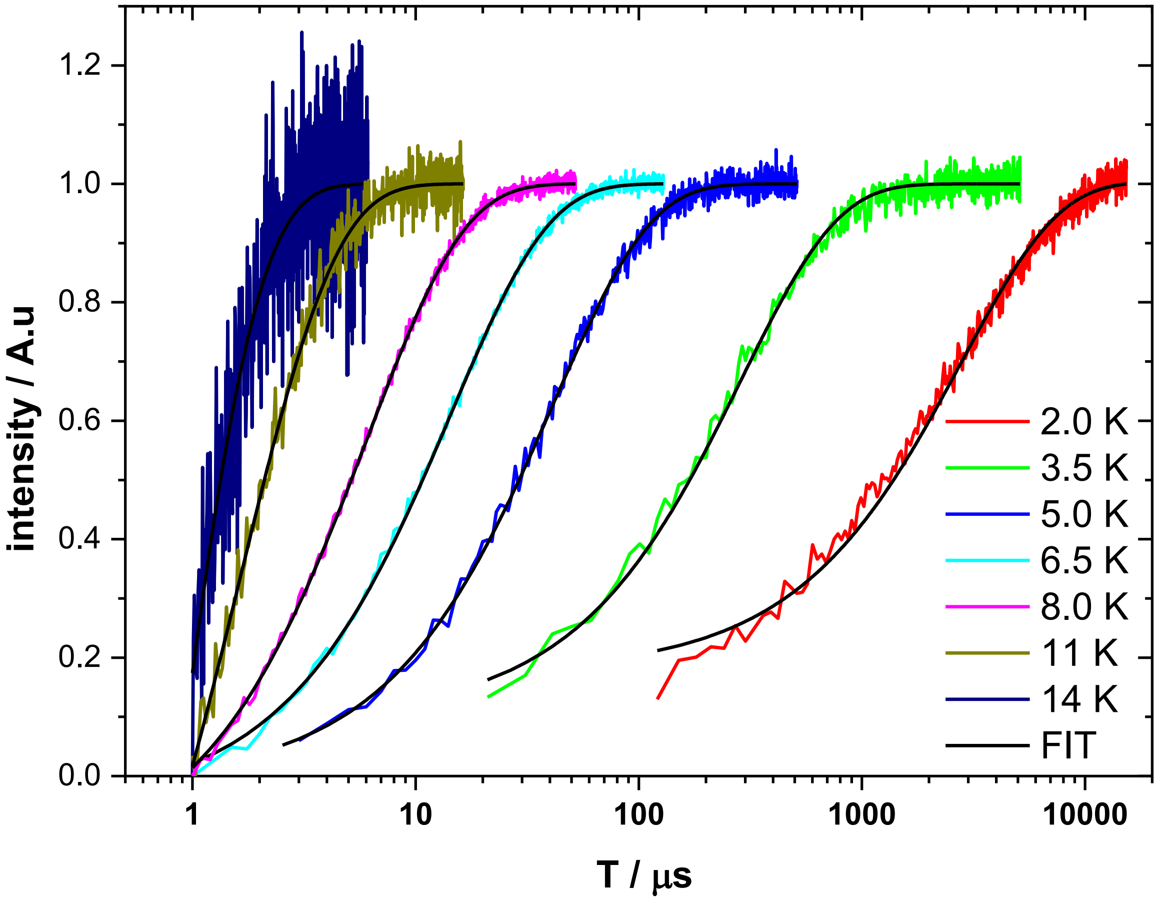

Figure S26. Inversion-recovery traces for transition **D** of **ΔYb** at 2671 Gauss. The experimental curves are vertically shifted and normalised to the equilibrium magnetisation at each temperature.

Table S7. T_1 values extracted from the data in Figures S13 to S16 .

T_1 / µs	2070 G	2109 G	2356 G	2671 G
2 K	2957.561(32.660)	2818.440(29.936)	2652616(29.262)	2795.346(31.189)
3.5 K	264.609(1.642)	255.149(1.275)	254.694(2.006)	287.267(3.216)
5 K	37.982(0.135)	36.244(0.100)	35.845(0.169)	41.489(0.304)
6.5 K	13.438(0.034)	12.217(0.036)	12.424(0.040)	14.508(0.052)
8 K	5.627(0.027)	5.314(0.018)	5.116(0.017)	6.109(0.023)
11 K	1.571(0.014)	1.459(0.011)	1.399(0.014)	1.627(0.020)
14 K	0.632(0.028)	0.593(0.020)	0.594(0.034)	0.669(0.038)

T_m time traces

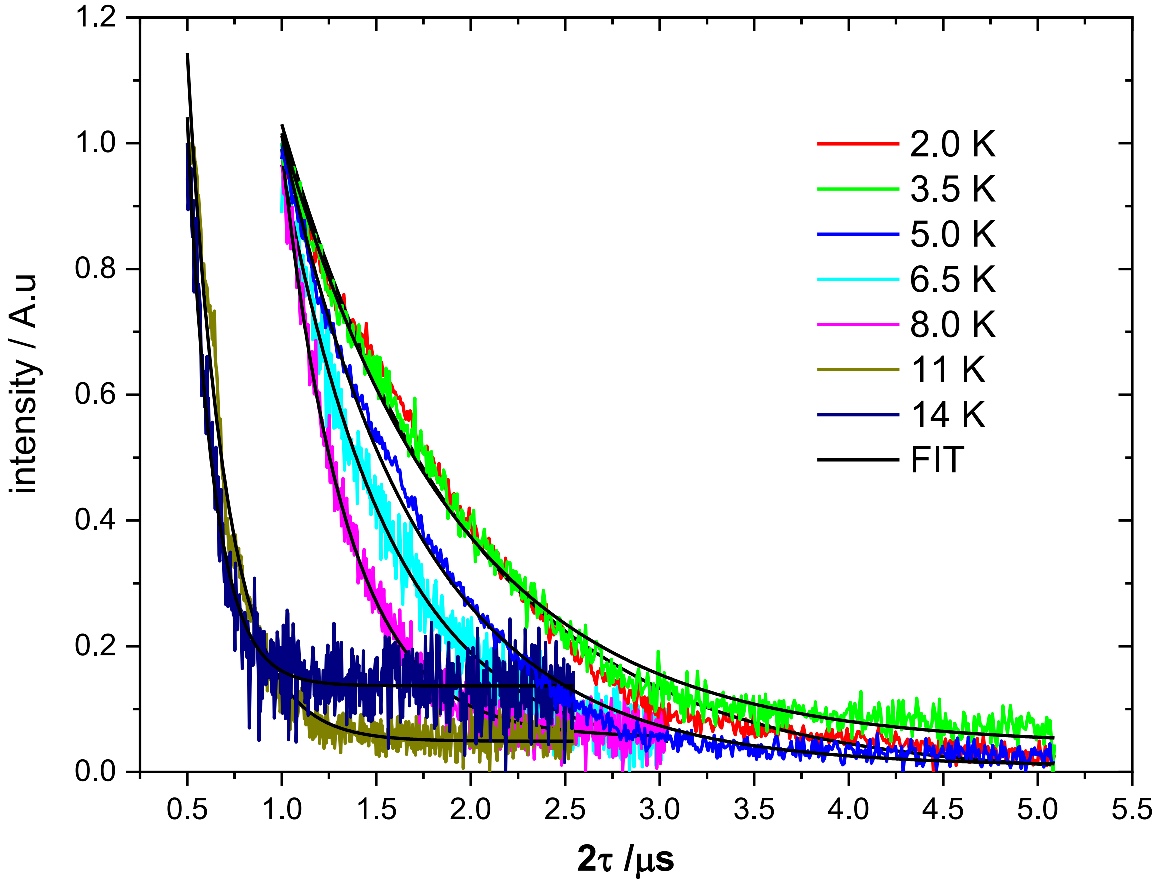

Figure S27. Normalised echo-decay traces for transition **A** of **ΔYb** at 2070 Gauss.

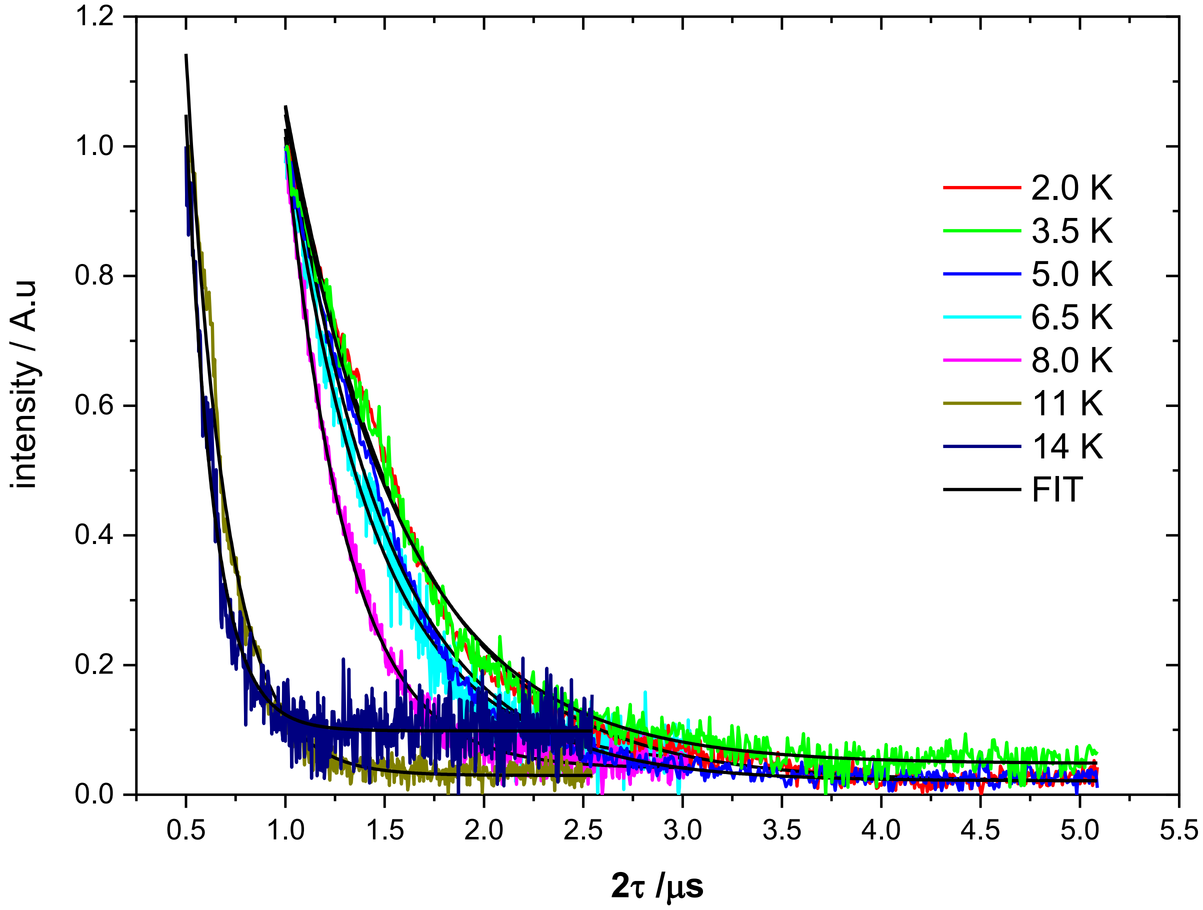

Figure S28. Normalised echo-decay traces for transition **B** of **ΔYb** at 2109 Gauss.

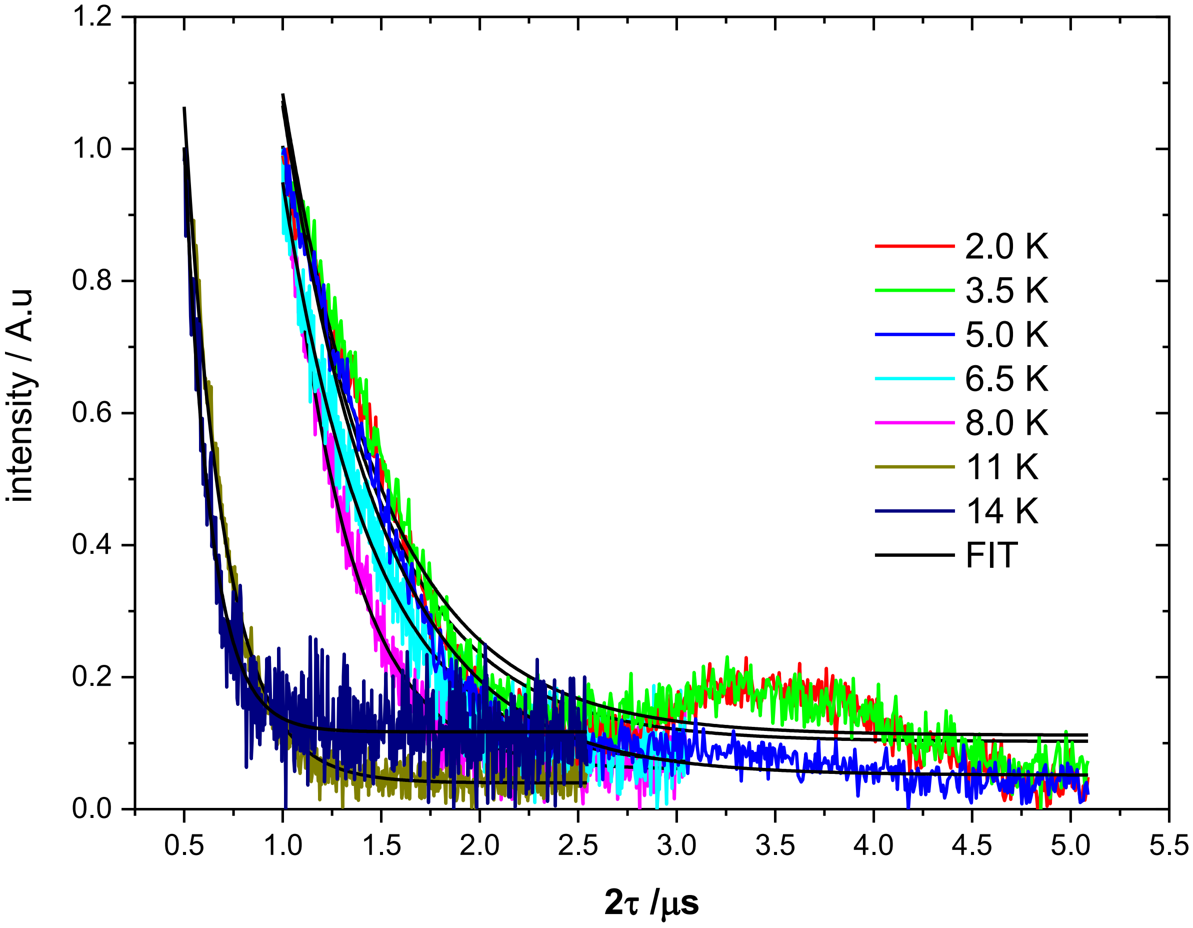

Figure S29. Normalised echo-decay traces for transition **C** of **ΔYb** at 2356 Gauss.

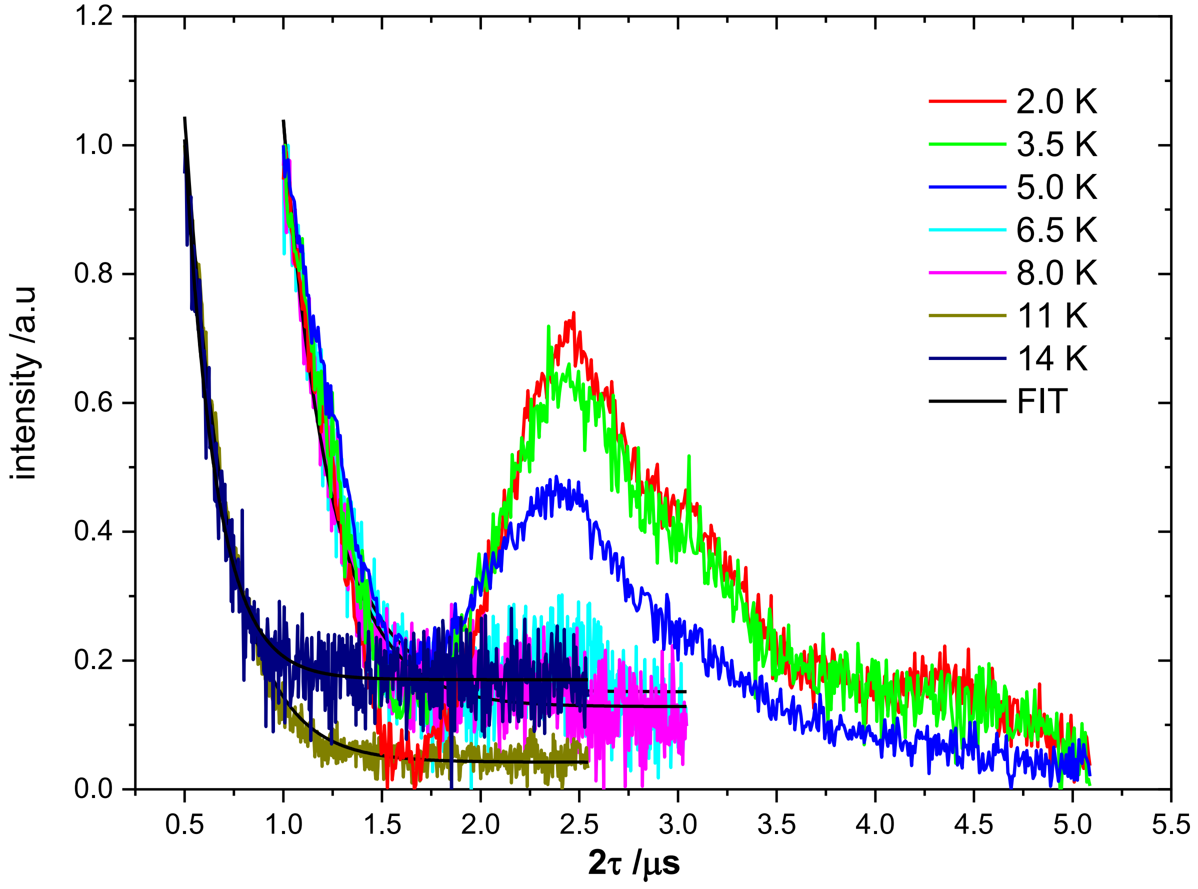

Figure S30. Normalised echo-decay traces for transition **D** of **ΔYb** at 2671 Gauss.

Table S8. T_2 values extracted from the data in Figures S17 to S20 .

T_2 / ns	2070 G	2109 G	2356 G	2671 G
2 K	997.4(10.1)	617.7(4.6)	504.3(11.7)	-
3.5 K	928.2(10.3)	588.4(5.8)	523.6(10.6)	-
5 K	727.8(5.6)	507.2(3.8)	512.6(6.3)	-
6.5 K	581.0(9.8)	452.0(6.5)	468.0(9.5)	240.0(7.3)
8 K	341.0(4.2)	302.0(2.5)	324.0(4.5)	240.7(5.5)
11 K	204.0(2.5)	201.0(2.1)	204.0(1.9)	218.3(1.9)
14 K	137.0(3.4)	137.6(2.9)	132.7(4.0)	159.2(4.1)

Rabi nutations

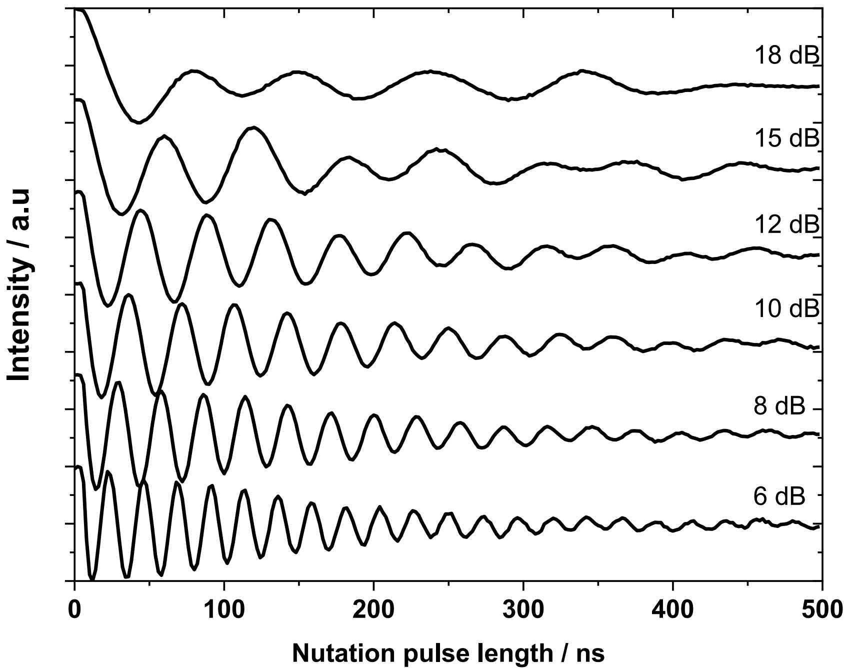

Figure S31. Rabi nutation for transtion **A** at 2070 Gauss and 5 K. Rabi frequencies have been determined by Fourier Transform of the transient nutation traces.

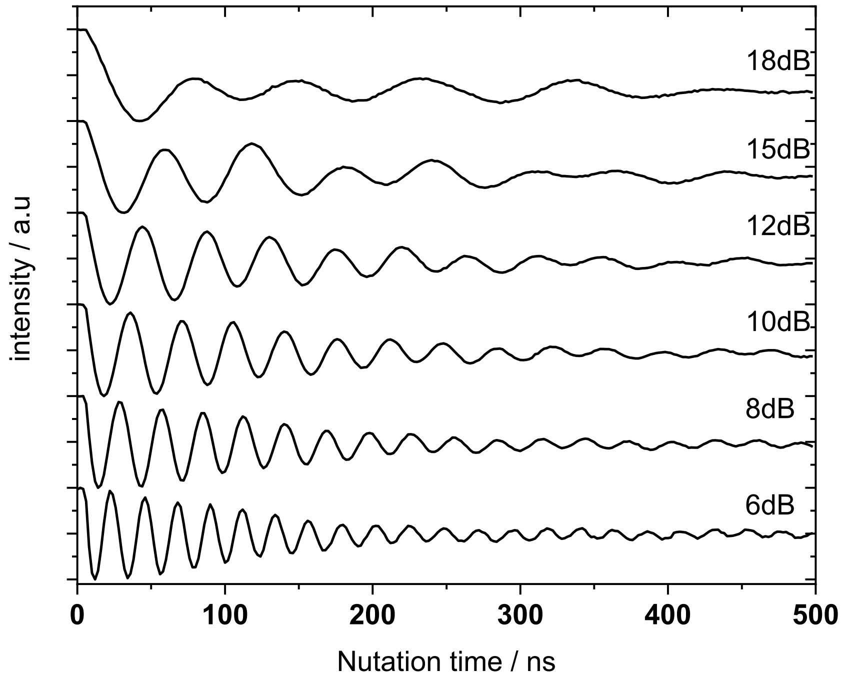

Figure S32. Rabi nutation for transtion **B** at 2109 Gauss and 5 K. Rabi frequencies have been determined by Fourier Transform of the transient nutation traces.

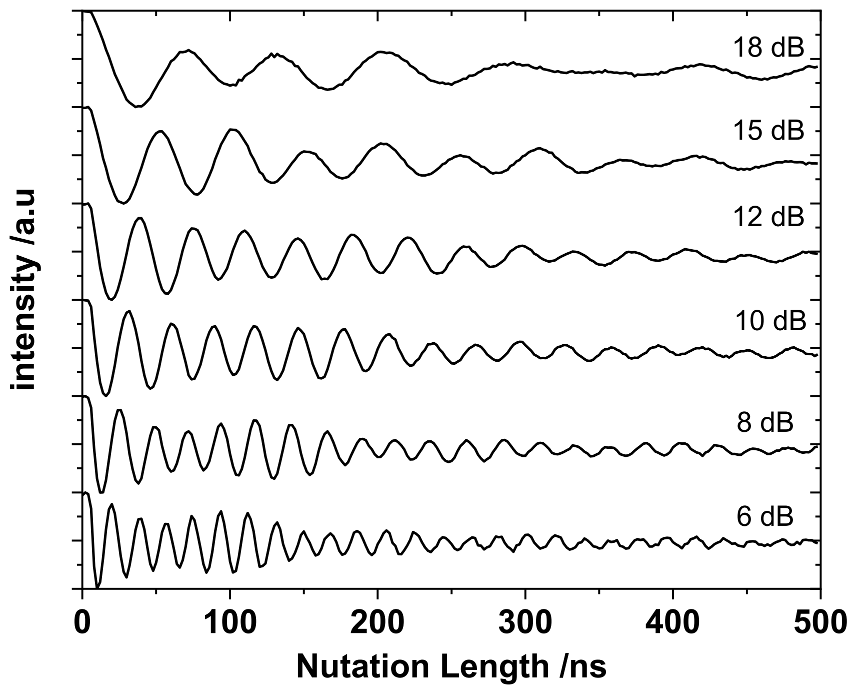

Figure S33. Rabi nutation for transtion **C** at 2356 Gauss and 5 K. Rabi frequencies have been determined by Fourier Transform of the transient nutation traces.

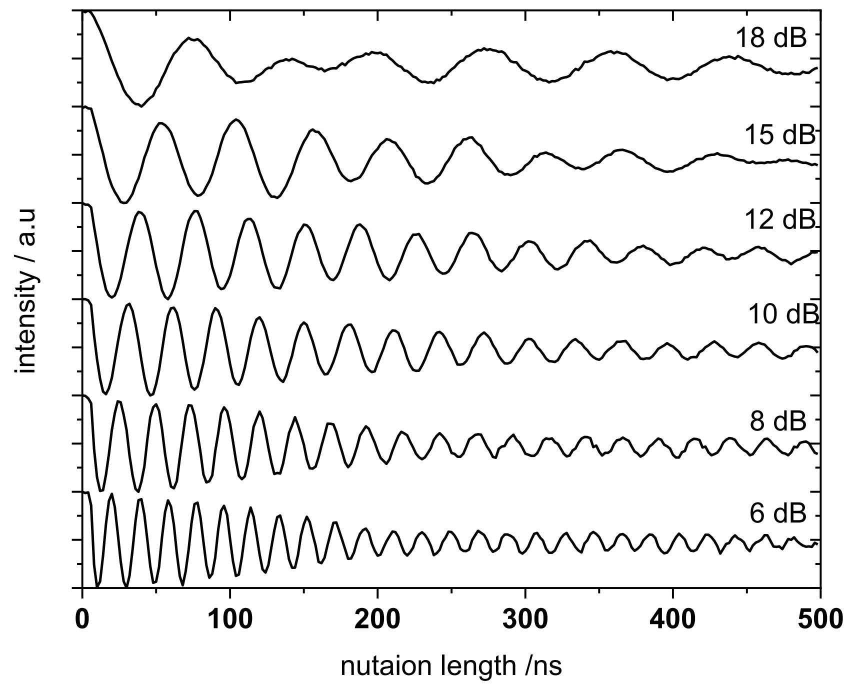

Figure S34. Rabi nutation for transtion **D** at 2671 Gauss and 5 K. Rabi frequencies have been determined by Fourier Transform of the transient nutation traces.

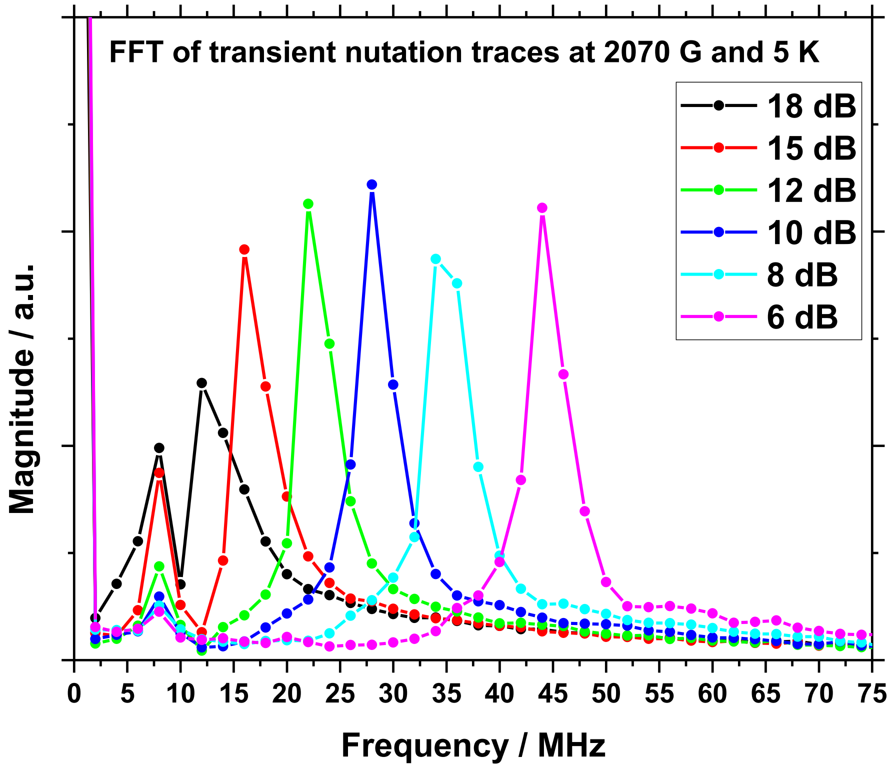

Figure S35. FFT of transient nutation traces for transtion **A** at 2070 Gauss and 5 K. The power independent frequency at about 8.8 MHz corresponds to the ^{1}H Larmor frequency.

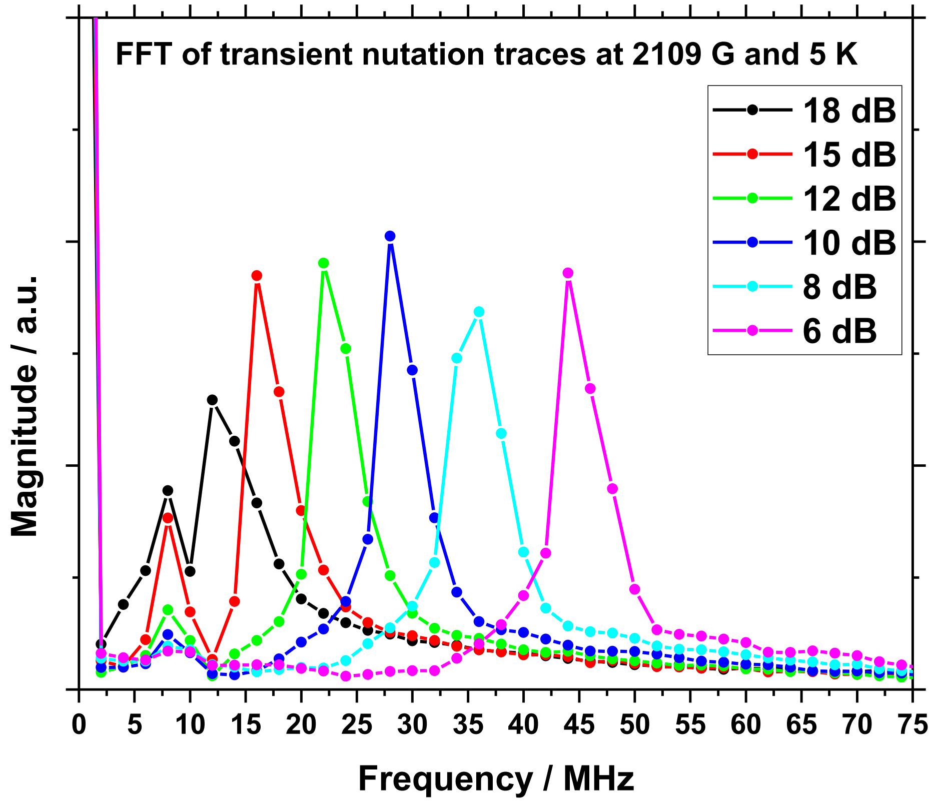

Figure S36. FFT of transient nutation traces for transtion **B** at 2109 Gauss and 5 K. The power independent frequency at about 9.0 MHz corresponds to the ^{1}H Larmor frequency.

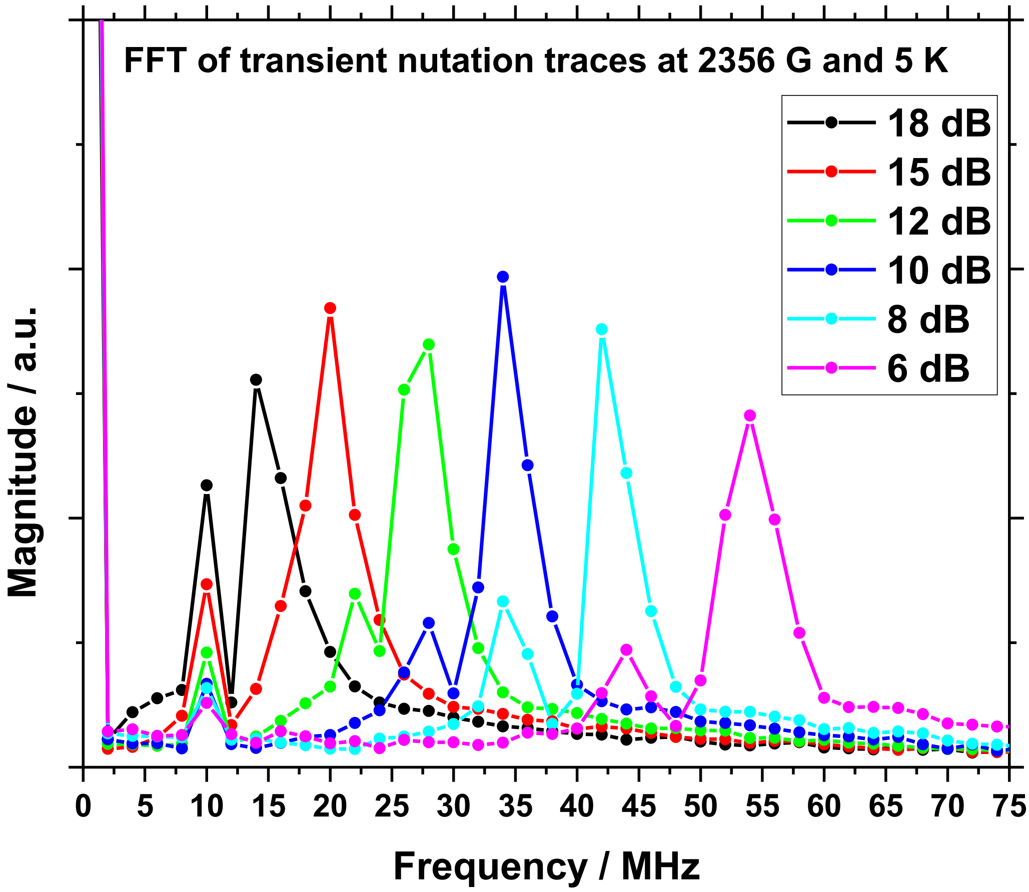

Figure S37. FFT of transient nutation traces for transtion **C** at 2356 Gauss and 5 K. The power independent frequency at about 10.0 MHz corresponds to the ^{1}H Larmor frequency.

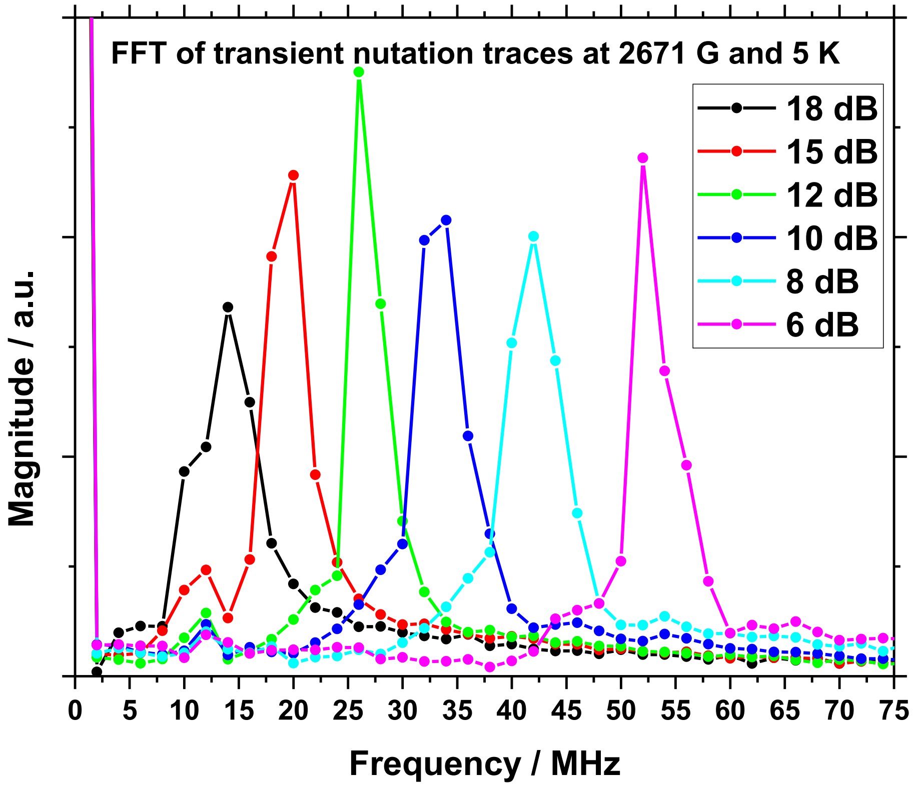

Figure S38. FFT of transient nutation traces for transtion **D** at 2671 Gauss and 5 K. The power independent frequency at about 11.4 MHz corresponds to the ^1H Larmor frequency.

EasySpin script for simulation of the Single Crystal EDFS

```
clear all; clc; clf;
%% Load Data

% To compare with experiment load in the comand below the experimental
% data.
% load('SI_single_crystal_data.mat');

%% Simulate with EasySpin

% Experimental
Exp.mwFreq=9.67713001229461;
Exp.Range=[140 340];
Exp.CrystalSymmetry='P212121';
Exp.MolFrame=[28.8387 13.7034 16.9812]*pi/180;
Exp.SampleFrame=[330.1702 71.6788 39.5116]*pi/180;
Exp.Harmonic=0;
Exp.nPoints=2000;

% Spin System
Sys.S=1/2;
Sys.g=[2.57 3.27 3.69];
Sys.gFrame=[0 0 0]*pi/180;
Sys.lwpp=[0.257831832667420 1.48578957699950];
Sys.Nucs='Yb';
Sys.A=[523 664 766];

% Simulation Function
[Field,Spec]=pepper(Sys,Exp);
Spec=rescaledata(Spec,spc,'lsq');
plot(B0,spc,Field,Spec)
```
